# Substrate specificity mapping of fungal CAZy AA3_2 oxidoreductases

**DOI:** 10.1186/s13068-024-02491-8

**Published:** 2024-03-27

**Authors:** Hongbo Zhao, Johanna Karppi, Owen Mototsune, Daria Poshina, Jenny Svartström, Thi Truc Minh Nguyen, Tri Minh Vo, Adrian Tsang, Emma Master, Maija Tenkanen

**Affiliations:** 1 Present Address: Department of Food and Nutrition, University of Helsinki, Helsinki, Finland; 2 Department of Chemical Engineering and Applied Chemistry, University of Toronto, Toronto, ON Canada; 3 Centre for Structural and Functional Genomics, Concordia University, 7141 Sherbrooke Street West, Montreal, QC H4B 1R6 Canada; 4 Department of Bioproducts and Biosystems, Aalto University, Espoo, Finland

**Keywords:** CAZy family AA3_2, Gentiobiose, Oxidoreductase, Phenoxy radical, Sequence similarity network

## Abstract

**Background:**

Oxidative enzymes targeting lignocellulosic substrates are presently classified into various auxiliary activity (AA) families within the carbohydrate-active enzyme (CAZy) database. Among these, the fungal AA3 glucose–methanol–choline (GMC) oxidoreductases with varying auxiliary activities are attractive sustainable biocatalysts and important for biological function. CAZy AA3 enzymes are further subdivided into four subfamilies, with the large AA3_2 subfamily displaying diverse substrate specificities. However, limited numbers of enzymes in the AA3_2 subfamily are currently biochemically characterized, which limits the homology-based mining of new AA3_2 oxidoreductases. Importantly, novel enzyme activities may be discovered from the uncharacterized parts of this large subfamily.

**Results:**

In this study, phylogenetic analyses employing a sequence similarity network (SSN) and maximum likelihood trees were used to cluster AA3_2 sequences. A total of 27 AA3_2 proteins representing different clusters were selected for recombinant production. Among them, seven new AA3_2 oxidoreductases were successfully produced, purified, and characterized. These enzymes included two glucose dehydrogenases (*Ta*GdhA and *Mc*GdhA), one glucose oxidase (*Ap*GoxA), one aryl alcohol oxidase (*Ps*AaoA), two aryl alcohol dehydrogenases (*As*AadhA and *As*AadhB), and one novel oligosaccharide (gentiobiose) dehydrogenase (*Ki*OdhA). Notably, two dehydrogenases (*Ta*GdhA and *Ki*OdhA) were found with the ability to utilize phenoxy radicals as an electron acceptor. Interestingly, phenoxy radicals were found to compete with molecular oxygen in aerobic environments when serving as an electron acceptor for two oxidases (*Ap*GoxA and *Ps*AaoA), which sheds light on their versatility. Furthermore, the molecular determinants governing their diverse enzymatic functions were discussed based on the homology model generated by AlphaFold.

**Conclusions:**

The phylogenetic analyses and biochemical characterization of AA3_2s provide valuable guidance for future investigation of AA3_2 sequences and proteins. A clear correlation between enzymatic function and SSN clustering was observed. The discovery and biochemical characterization of these new AA3_2 oxidoreductases brings exciting prospects for biotechnological applications and broadens our understanding of their biological functions.

**Supplementary Information:**

The online version contains supplementary material available at 10.1186/s13068-024-02491-8.

## Background

Auxiliary activities (AA) redox enzymes have been catalogued in the carbohydrate-active enzyme (CAZy) database since 2013 [1]. In the CAZy database, the AA3 family of enzymes belong to the glucose–methanol–choline (GMC) oxidoreductase superfamily [2]. These AA3s are flavoproteins that carry flavin adenine dinucleotide (FAD) or its derivatives as a co-factor, either covalently or non-covalently linked. They can utilize several electron acceptors, including various quinones, phenoxy radicals, and metal ions [3]. The AA3 is divided into four subfamilies, of which subfamilies 1, 3, and 4 contain cellobiose dehydrogenases, alcohol oxidases, and pyranose oxidases, respectively. Subfamily 2, known as AA3_2, is the most diverse in terms of electron donor and electron acceptor specificity, encompassing aryl alcohol oxidases/dehydrogenases (AAOs/AADHs; EC 1.1.3.7), and carbohydrate-active enzymes such as pyranose dehydrogenases (PDHs; EC 1.1.99.29), glucose oxidases (GOxs; EC 1.1.3.4), glucose dehydrogenases (GDHs; EC 1.1.5.9), and oligosaccharide dehydrogenases (ODHs; EC 1.1.5.–) [4, 5]. While GOxs, GDHs, and ODHs specifically oxidize the anomeric carbon of carbohydrates, forming the corresponding lactones that spontaneously hydrolyse to carboxylic acid in water, PDHs demonstrate a broader regioselectivity, oxidizing the secondary hydroxyls from the C2 to C4 positions into ketones and, in some cases, the anomeric carbon [6–9].

The AA3_2 enzymes have shown considerable promise in various biotechnological and biomedical applications. For instance, AAOs have been used for the synthesis of flavours, fragrances, and other high-value biochemicals [10–12]. They also show potential for dye decolorization and pulp bio-bleaching by delivering hydrogen peroxide [13, 14]. PDHs, which can introduce multiple carbonyls to a single sugar molecule, can serve as a green catalyst to generate bio-based cross-linkers and high-value biochemicals [9, 15]. AA3_2 GOx and GDH enzymes are excellent candidates for glucose-oxidizing enzymatic anodes to use in enzymatic biological fuel cells that generate electricity [16–19]. In addition, GOxs have been used in the biosensor of blood glucose meters for diabetes self-monitoring, and GDHs, being independent of oxygen levels, have gained interest for similar applications [20]. Moreover, previous research has explored the use of GOx and hemoglobin for in vitro tumour destruction by targeting the delivery of GOx and haemoglobin to initiate nutrient starvation and the cytotoxic Fenton reaction around cancer cells, representing a potential approach for future cancer treatment [21].

The significance of AA3 activities in the direct and indirect microbial degradation of plant biomass, as well as in the modification of plant and microorganism cell walls through enzyme interactions, has become increasingly apparent over the past decade. For instance, AA3_2 carbohydrate oxidoreductases are believed to synergize with lytic polysaccharide monooxygenases (LPMOs) for the oxidative degradation of cellulose [22]. Additionally, AA3_2 aryl alcohol oxidase has been employed in enzyme cascade alongside lignin peroxidase for lignin depolymerization [23]. In addition to degrading plant cell walls, one study suggested that an AA3_2 carbohydrate oxidase from the plant pathogen *Ustilago maydis* participates in the modification of its own fungal cell wall via the interplay with a glycoside hydrolase [24]. The interplay of AA3_2 AADH, GDH, and laccases was also observed, and both AA3_2 AADH and GDH were found to inhibit the formation of laccase-oxidized phenolic products [25, 26]. Moreover, a recent genetic study has revealed that large numbers of copies of AA3_2 encoding genes of Basidiomycota fungi co-occur with large numbers of copies of AA2 class II high-redox peroxidases, particularly in white rot and litter decomposing fungi, suggesting a possible role for AA3_2s in lignin oxidation and degradation [27].

Even though the CAZy database holds more than 2400 predicted AA3_2 sequences, only a few dozen AA3_2 proteins have been biochemically characterized to date. The majority of characterized AA3_2 sequence clusters fall into two main clades: one comprising phylogenetically related PDHs and AAO/AADHs, and the other comprising GDHs and GOxs [4]. The PDH-AAO/AADH clade further divides into three subclades, AAO, AAO-like, and PDH [4]. With members such as *Pe*AAOx from *Pleurotus eryngii* and *Am*PDH1 from *Leucoagaricus meleagris* (syn. *Agaricus meleagris*) being biochemically and structurally characterized in the AAO and PDH subclades [8, 28], it is believed that the sequences within the AAO-like subclade exhibit a transitional architecture, possessing the structural elements of both AAOs and PDHs, and possibly having an extended substrate specificity that might oxidize both sugars and alcohols. However, no characterized members of the AAO-like subclade are available to confirm this hypothesis [4]. The GDH-GOx clade segregates into four major subclades, GOx I, GDH I, GDH II, GDH III, and a minor subclade, GOx II [4, 29]. Enzymes within subclades GOx I and GDH I are biochemically and structurally characterized, and they were found to be rather specific towards glucose [30, 31]. In contrast, the characterized enzymes within the subclades GOx II, GDH II, and GDH III displayed broader substrate specificity and they were recently found to have high kinetic efficiency towards disaccharides, including laminaribiose, gentiobiose, and maltobiose [5, 29]. However, the combination of the PDH-AAO clade and GDH-GOx clade only covers around 25% of total fungal AA3_2 sequences, leaving a substantial portion still unclassified. To date, only two aryl alcohol oxidases have been characterized from the AA3_2 sequence space outside the area of these two clades [32, 33].

Recognizing the limitations in the current understanding of AA3_2 enzymes, we conducted sequence similarity network (SSN) analyses of fungal AA3_2 sequences. The SSN is used as an alternative to traditional phylogenetic trees because the SSN can be easily visualized and used to predict sequence–function relationships. Simultaneously, we performed the recombinant production and characterization of new fungal AA3_2 proteins from five SSN clusters. These AA3_2s were screened with three electron acceptors (benzoquinone [BQ], dichlorophenolindophenol [DCIP], and oxygen) over 15 substrate mixtures with 52 distinct substrates. Subsequently, the activities detected on the substrate mixtures were further resolved with individual substrates, and in-depth analyses of the AA3_2s were conducted with the best oxidized substrates. The biochemical analyses of the produced proteins revealed new enzymes to oxidize aryl alcohol, monosaccharides, and disaccharides. In particular, a novel oligosaccharide dehydrogenase (*Ki*OdhA) was identified, with gentiobiose being primarily oxidized. Notably, the AA3_2 enzymes were found to use phenoxy radicals as election acceptors.

## Results

### Protein specificity dependent clustering of fungal AA3_2s

**Fig. 1:**
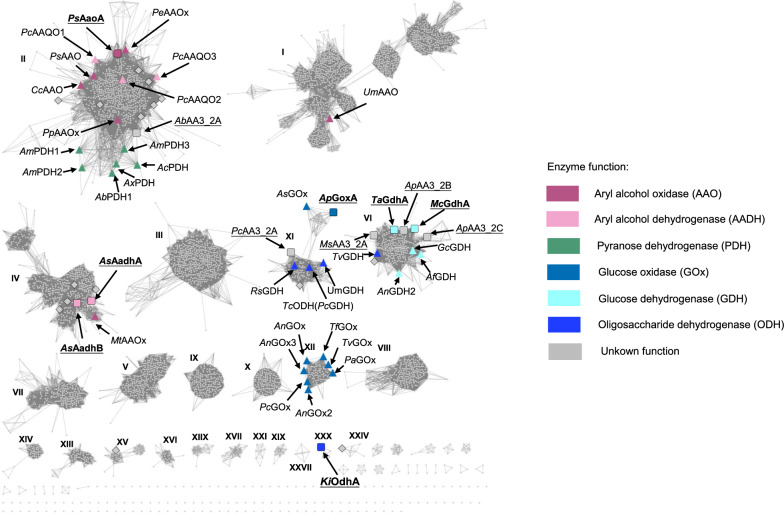
Sequence similarity network at a bit-score cut-off of 420 for fungal sequences. The previously biochemically characterized sequences (details in Additional file 1: Table S1) and the sequences selected by this study (details in Additional file 1: Table S3) are shown with large symbols, while the remaining sequences are represented by grey dots. The sequences were colored based on specificity, and the shape reveals the characterization status (triangle: previously characterized; diamond: failed in production; square: successful production). The successfully produced enzymes with characterized activities are bolded and underlined, while the produced proteins without any detected activity are solely underlined. Enzyme function and naming is according to results in this study

The AA3_2 proteins used in this study included 4450 AA3_2s from published fungal genomes in MycoCosm (https://mycocosm.jgi.doe.gov/mycocosm/home), 7 biochemically characterized AA3_2s from MycoCLAP [34], and 304 AA3_2s from the Centre for Structural and Functional Genomics (CSFG) at Concordia University. After removing identical sequences, the final AA3_2 collection consisted of 4577 AA3_2 protein sequences and 4578 AA3_2 domain sequences. In addition, a set of 30 biochemically characterized protein sequences from fungi (Additional file 1: Table S1) was added to our fungal AA3_2 putative sequence dataset, in which 17 were found to be redundant. The remaining 13 sequences were included in our final sequence dataset (4590 sequences in total). SSN, based on pairwise alignment of sequences, was used to cluster sequences based on their homology. The statistical significance of the alignment score was represented as bit-score, a higher bit score indicates better alignment. In a SSN, nodes represent sequences, and linkages between nodes indicate significant similarity between sequences. These linkages are determined using bit-score as threshold, where sequences with bit-score above the threshold are connected. By labelling the previously identified AA3_2 sequences in the SSN, we were able to alter the stringency of this threshold, thereby mapping the various enzymatic activities to separate clusters (Fig. 1). The cluster numbers were assigned based on the number of individual sequences within each cluster, starting from cluster I, which has the most sequences, and descending to clusters with fewer sequences (Additional file 1: Table S2). Specifically, with a bit-score of 420, cluster II, the second largest cluster in the SSN, comprises solely pyranose dehydrogenases and aryl alcohol oxidases/dehydrogenases, which are drawn to the cluster’s two sides. With a higher cut-off and bit-score of 470, cluster II can be again divided into two subclusters, with AAOs/AADHs in subcluster IIa and PDHs in subcluster IIb (Additional file 1: Fig. S1). Aryl-alcohol-oxidizing activities were also found in clusters I and VI, whereas the carbohydrate-oxidizing activities were identified in clusters VI, XI, and XII. Interestingly, oligosaccharide-oxidizing activities were found in clusters VI and XI, while cluster XII contains solely glucose oxidases.

**Fig. 2:**
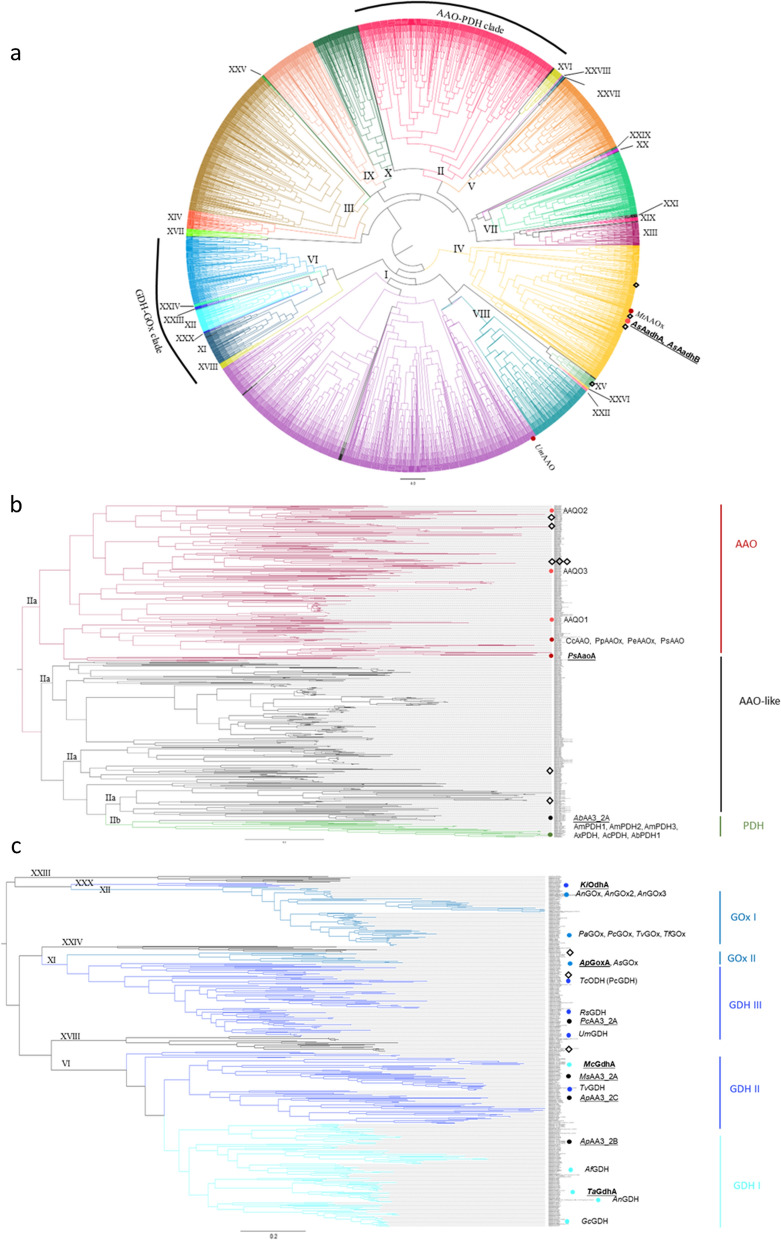
The maximum likelihood trees of fungal AA3_2 sequences. **a** The maximum likelihood tree of all collected fungal AA3_2 sequences in this study. **b**, **c** Maximum likelihood trees of the AAO-PDH clade (**b**) and GOx-GDH clade (**c**). The phylogenetic clades that were defined by [4] are shown on the right side of the figure. The characterized proteins and the proteins that were produced successfully in this study are marked as solid circles. The proteins that were not successfully produced are marked as diamonds. The position of proteins within the AAO-PDH and GOx-GDH clades are indicated in Fig. 2b, c while the position of proteins outside the AAO-PDH and GOx-GDH clades are indicated in Fig. 2a. The sequences within the top 30 clusters in the SSN are highlighted in the branches

To further inform the sequence selection, maximum likelihood trees were generated of the fungal AA3_2 sequences. As can be seen in Fig. 2a, the previously characterized AA3_2 members are mainly distributed within two clades of the tree, which was defined by Sützl in 2018 as the AAO-PDH and GDH-GOx clades [3]. The sequences within the AAO-PDH clade correspond to the sequences in SSN cluster II, and the sequences within the GDH-GOx clade correspond to the members in clusters VI, XI, and XII. Additionally, members of the clusters XVIII, XXIII, XXIV, and XXX in the SSN had a high level of homology to the members of the GDH-GOx clade, as shown in the maximum likelihood tree (Fig. 2a). Therefore, we took the members of the SSN cluster II and the members of the SSN clusters XI, VI, XII, XVIII, XXIII, XXIV, and XXX for the generation of two distinct trees (Fig. 2b, c).

Guided by the SSN and maximum likelihood trees, 27 fungal AA3_2 proteins were selected for production, while the selection tried to maximize the coverage of fungi species and lifestyle, with 19 fungal species from both Ascomycota and Basidiomycota covering several ecologies and habitats (Additional file 1: Table S3). Specifically, nine proteins were chosen from the SSN cluster II, with six of them belonging to the AAO clade and three of them belonging to the AAO-like clade. Nine proteins were selected from clusters VI and XI, with most of the GDH-GOx subclades covered. In addition, we chose three extra proteins with no known activities from clusters XV, XXIV, and XXX.

### Protein production and primary activity screen

Out of the 27 selected proteins, 12 were successfully produced in *P. pastoris* (Table 1). Instead of trying to optimize the transformation and to express the remaining 15 proteins, we decided to focus on the proteins that were successfully produced as they covered most of the clusters of interest. A uniform nomenclature for bacterial proteins was followed in this study where the source organism of each protein is represented by the first capital letter of the genus name and the first character of the species name [35]. Once a protein is experimentally characterized, a name is then assigned based on its enzymatic function with the first letter capitalized (Table 1). The proteins without known activity are represented by AA3_2, while paralogs are denoted by a single uppercase letter.

**Table 1:** Proteins successfully produced in *Pichia pastoris* KM71H

SSN cluster	Tree clade	Protein	Source species	CBM	FAD	Predicted mass (kDa)
IIa	AAO-Like	*Ab*AA3_2A	*Agaricus bisporus var bisporus*	N	Y	62.6
IIa	AAO	*Ps*AaoA	*Punctularia strigosozonata*	N	Y	63.3
IV	n.a	*As*AadhA	*Auricularia subglabra*	1	Y	72.4
IV	n.a	*As*AadhB	*Auricularia subglabra*	1	Y	74.7
VI	GDH II	*Ap*AA3_2B	*Aureobasidium pullulans*	N	Y	64.0
VI	GDH I	*Ap*AA3_2C	*Aureobasidium pullulans*	N	N	64.7
VI	GDH II	*Mc*GdhA	*Malbranchea cinnamomea*	N	Y	64.7
VI	GDH II	*Ms*AA3_2A	*Myceliophthora sepedonium*	N	Y	63.4
VI	GDH I	*Ta*GdhA	*Thermoascus aurantiacus*	N	Y	62.7
XI	GOx II	*Ap*GoxA	*Aureobasidium pullulans*	N	Y	65.0
XI	GDH III	*Pc*AA3_2A	*Phanerochaete carnosa*	N	Y	66.9
XXX	n.a	*Ki*OdhA	*Kockovaella imperatae*	13	Y	78.4

*AAO* aryl alcohol oxidase, *AADH* aryl alcohol dehydrogenase, *CBM* cellulose binding module, *GOx* glucose oxidase, *GDH* glucose dehydrogenase, *n.a.* not available, *ODH* oligosaccharide dehydrogenase

Purification was achieved with immobilized metal affinity chromatography. The characteristic peak for the presence of the oxidized FAD was seen at about 375–440 nm for *Ab*AA3_2A, *Ps*AaoA*, Ap*GoxA, *As*AadhA, *As*AadhB, *Mc*GdhA, *Ms*AA3_2A, *Ta*GdhA, and *Ki*OdhA (Additional file 1: Fig. S2). However, the oxidized FAD could not be clearly seen for *Ap*AA3_2C; consequently, exogenous FAD was added to *Ap*AA3_2C for the subsequent activity assays. The *Pc*AA3_2A and *Ap*AA3_2B stock concentrations (2.2 and 2.4 mg/ml, respectively) were below the spectroscopy-based FAD detection threshold. Partial degradation of *Ab*AA3_2A and *Ap*AA3_2B was observed; all AA3_2 proteins appeared to be glycosylated based on showing higher electrophoretic molecular weight than what was predicted from corresponding protein sequences (Additional file 1: Fig. S3). The substrate specificity of the purified proteins was screened with 52 distinct electron donors (substrates) and three electron acceptors: BQ, DCIP, and oxygen. The selection of substrates was guided by a list of substrates previously known to be oxidized by AA3 enzymes. The list served as a foundation upon which we expanded to include additional substrates with similar chemical properties and functional groups, covering monosaccharides; disaccharides with different linkages; primary, secondary, and aryl alcohols; and polyols (Additional file 1: Table S4). The substrates with commonality in their chemical structures were pooled together into 15 substrate mixtures. The three electron acceptors were selected for two reasons: First, our study aimed to investigate whether the enzymes could utilize oxygen as an electron acceptor. Second, we sought to determine if the enzymes could function as dehydrogenases. Based on previous research, BQ and DCIP are among the most effective electron acceptors for fungal AA3 enzymes. The activity screening was first conducted on the substrate mixtures with each electron acceptor in multi-well plates by colorimetric assay. This approach allowed us to efficiently screen a wide range of potential electron donors while minimizing the number of experiments required.

**Fig. 3:**
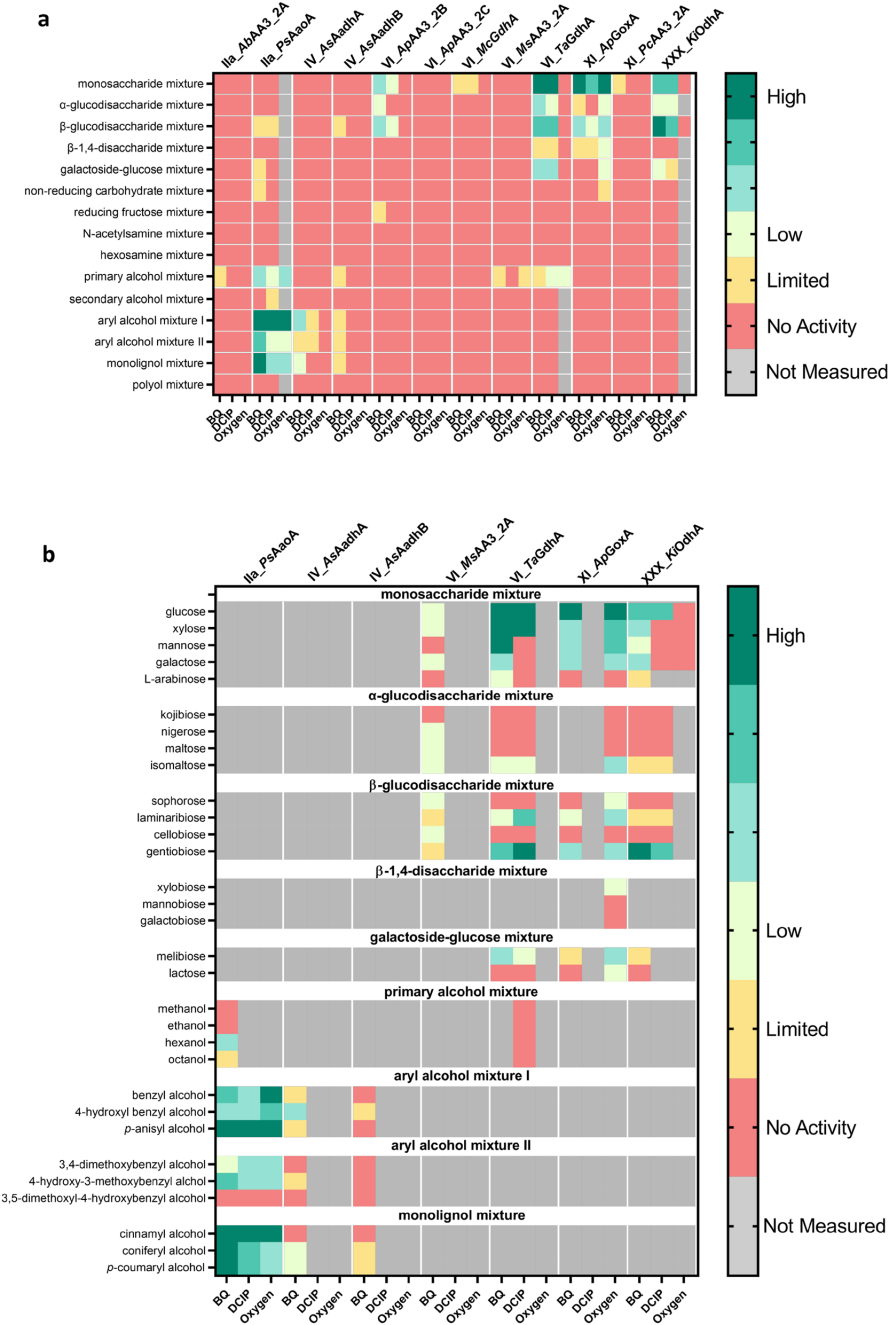
Activity heat map of fungal AA3_2 proteins at pH 5 and 30 ℃. **a** The first-level screening on the substrate mixtures. **b** The second-level screening of individual substrates that were oxidized in the first-level mixture. Oxidase activity was assessed by coupling the H_2_O_2_ production to the oxidation of 2,2′-azinobis (3-ethylbenzthiazoline-6-sulfonic acid) (ABTS) by horseradish peroxidase (HRP), while dehydrogenase activity was determined using BQ and DCIP as electron acceptors. The levels of the activity were defined based on the measured activity. High: > 1 U/mg; medium: 0.2–1 U/mg; moderate: 0.01–0.2 U/mg; and low: quantifiable—0.01 U/mg. Limited activity signified a minimum 10% difference in the absorbance between the reaction and the background after overnight incubation. (The activities of *Pc*AA3_2A and *Ap*AA3_2B’s were later proved to be false positives.)

A cluster–substrate specificity correlation was observed from the initial screening. Members of clusters IIa and IV oxidized aryl alcohols, whereas members of clusters VI, XI, and XXX oxidized carbohydrates (Fig. 3a). To provide a qualitative indication of enzymatic activities, we defined six levels of activity, ranging from high to limited (Fig. 3). Specifically, *Ps*AaoA (cluster IIa) was found to be highly active with aryl alcohols and monolignols, while *As*AadhA (cluster IV) displayed low activity with them. The enzymes of clusters VI and XI, including *Ap*AA3_2B, *Ta*GdhA, and *Ap*GoxA, showed high activity with monosaccharides, while *Ki*OdhA from cluster XXX had high activity with the β-glucodisaccharide mixture, and moderate activity with the monosaccharide mixture. Notably, *Ps*AaoA (cluster IIa) and *Ap*GoxA (cluster XI) both displayed high oxidase activities, whereas the enzymes in clusters IV, VI, and XXX displayed only dehydrogenase activity. The reactions were left overnight at room temperature and measured again to detect minimal activities. Based on the values taken on the second day, *Ab*AA3_2A and *Ms*AA3_2A showed minimal possibility for oxidizing primary alcohols. *Mc*GdhA and *Pc*AA3_2A displayed limited activity with the monosaccharide mixture. *Ab*AadhB exhibited activity in a trace amount on aryl alcohol and monolignol mixtures. However, these activities could not be reliably confirmed by colorimetric assay alone. *Ap*AA3_2C showed no activity on any of the tested substrates, even with added FAD. All proteins with limited to no activity at pH 5 were screened again at pH 7, but no clear differences were observed (data not shown). For the proteins where no activity was detected, we anticipate several possible reasons. These include incorrect incorporation of FAD, improper protein folding, or the possibility that the true electron donor or acceptor was not included in the substrate mixture used in our screening assay.

After the initial activity screening with mixtures, more detailed analyses were conducted on individual substrates for enzymes exhibiting at least low levels of activity, along with their corresponding electron acceptors (Fig. 3b). Despite its limited activity, *Ab*AadhB was also assessed on the individual aryl alcohols due to its close homology with *Ab*AadhA (68%). *Ps*AaoA (cluster IIa) demonstrated activity with all three tested electron acceptors, exhibiting high activity with cinnamyl alcohol and p-anisyl alcohol. It also showed low activity with hexanol. Conversely, both *As*AadhA and *As*AadhB (cluster IV) showed a preference for 4-hydroxybenzyl alcohol, coniferyl alcohol, and p-coumaryl alcohol using only BQ as an electron acceptor but exhibited no action towards cinnamyl alcohol.

The carbohydrate-oxidizing enzymes possessed distinct oxidation profiles (Fig. 3b). *Ta*GdhA (cluster VI) showed high activity with glucose, xylose, and mannose. Additionally, *Ta*GdhA displayed moderate activity towards gentiobiose. The choice of electron acceptor influenced the enzyme activity. *Ta*GdhA exhibited good oxidation of mannose when using BQ as the electron acceptor, but no activity was detected on mannose when DCIP was the electron acceptor. Conversely, gentiobiose was oxidized at a higher rate by *Ta*GdhA with DCIP than with BQ at pH 5 and 30 °C. *Ap*GoxA (cluster XI) interestingly accepted both oxygen and BQ, while DCIP was not preferred. *Ap*GoxA showed high specificity towards glucose. Other monosaccharides and disaccharides were oxidized much less. A novel dehydrogenase, *Ki*OdhA, was discovered from cluster XXX and displayed high activity towards gentiobiose. It showed medium activity on glucose and some on xylose and galactose. *Ap*AA3_2B (cluster VI) exhibited only low to no activity on all of the tested monosaccharides and disaccharides. The proteins with activity detected at the screening stage were subjected for optimum pH characterization, kinetic analysis, product identification and quantification.

### pH profile

**Fig. 4:**
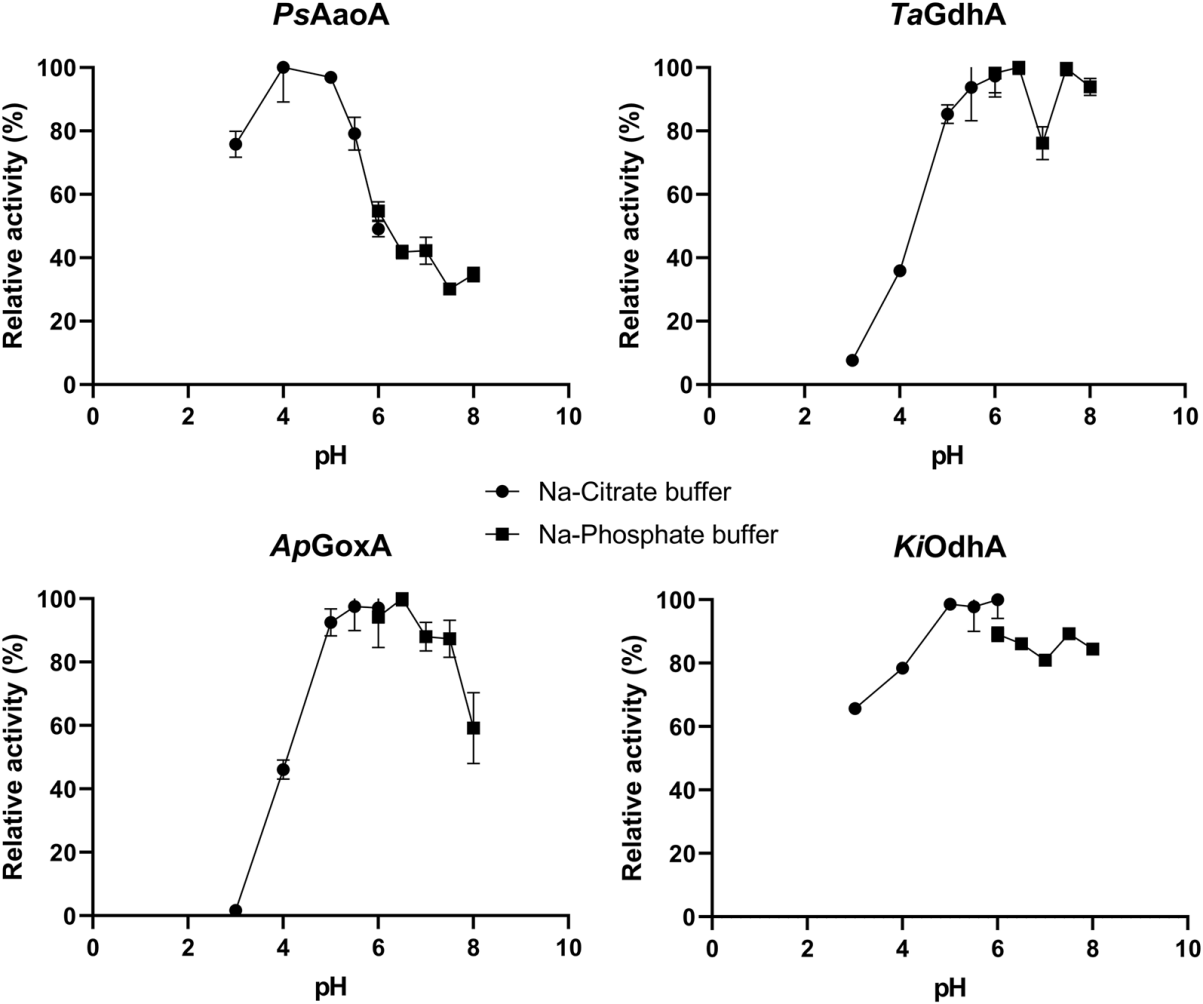
pH profiles of the four AA3_2s with high levels of activity. BQ was used as the electron acceptor. *Ps*AaoA was assayed with p-anisyl alcohol, *Ta*GdhA and *Ap*GoxA were assayed with glucose, and *Ki*OdhA was assayed with gentiobiose. Error bars represent the standard deviation of three replicated reactions

The effect of pH on the activity of *Ps*AaoA, *Ta*GdhA, *Ap*GoxA, and *Ki*OdhA was examined with their preferred electron donors, using BQ as the electron acceptor (Fig. 4). Other enzymes were not included in the pH profiling because their detected activities were too low or below the quantification limit. The carbohydrate-oxidizing enzymes *Ap*GoxA, *Ta*GdhA, and *Ki*OdhA showed a broad pH profile, with the optimal pH ranging from pH 5.0 to pH 7.5 (Fig. 4). Activity dropped rapidly below pH 5 for *Ta*GdhA and *Ap*GoxA, whereas *Ki*OdhA showed better tolerance of a harsh pH, maintaining more than 65% of its maximum activity between pH 3.0 and pH 8.0. The aryl-alcohol-oxidizing enzyme *Ps*AaoA displayed a narrower pH profile, with the most activity measured between pH 4.0 and pH 5.0. Activity was below 50% of the maximum activity at pH values higher than pH 6.0. Based on the pH profile, pH 5.5 was chosen for the subsequent studies for *Ta*GdhA, *Ap*GoxA, and *Ki*OdhA, and pH 5.0 was chosen for *Ps*AaoA. Because the specific activities were insufficient to determine a reliable pH optimum for *As*AadhA, *As*AadhB, and *Ap*AA3_2B, the extended product identification was carried out at pH 5.0, where their activities were initially detected.

### Product identification

Two separate methods were established to follow the reaction and evaluate product formation and the degree of oxidation. The oxidation of aryl alcohols was investigated using an ultra-high-performance liquid chromatography-photodiode array (UPLC-PDA) method, with which compounds were identified based on distinct retention times and the UV/visible light spectra (Additional file 1: Table S5). The oxidation of carbohydrates was evaluated using mass spectrometry in the negative mode.

*Ps*AaoA, *As*AadhA, and *As*AadhB oxidized the aryl alcohols to corresponding aldehydes, as confirmed by UPLC-PDA. For instance, coniferyl alcohol eluted at retention time (RT) 4.487 min, and after *Ps*AaoA oxidation for 8 h, a new peak with a RT of 6.839 min was detected, which had the identical UV/visible spectrum and retention time as the coniferyl aldehyde standard (Additional file 1: Fig. S4). All of the studied carbohydrate-acting enzymes oxidized the reducing end C-1. No oxidation of other positions was identified. *Ta*GdhA and *Ap*GoxA both oxidized glucose to gluconic acid (Additional file 1: Fig. S5). Glucose was detected as the chlorine adduct [M + Cl]^−^ at a mass-to-charge ratio of 215. The oxidation at the reducing anomeric centre resulted in the formation of glucolactone, which is not stable in water and hydrates to gluconic acid, with a mass-to-charge ratio of 195 [M−H]^−^. No other clear peaks were identified in the mass spectra. Neither *Ap*GoxA nor *Ta*GdhA were able to oxidize methyl glucose (results not shown), indicating that both enzymes oxidize carbohydrates solely at the reducing end C-1. Similarly, *Ki*OdhA was found to oxidize gentiobiose to gentiobionic acid (Additional file 1: Fig. S6). Gentiobiose was detected with a mass-to-charge ratio of 377 [M + Cl]^−^ and gentiobionic acid was detected with a mass-to-charge ratio of 357 [M−H]^−^. Surprisingly, no oxidation products were identified by MS for *Ap*AA3_2B and *Pc*AA3_2A on any of the tested substrates (glucose, xylose, galactose, nigerose, maltose, isomaltose, sophorose, and cellobiose), indicating that the screening by colorimetric assay may have shown false positive results (Additional file 1: Fig. S7). *Mc*GdhA was determined to oxidize glucose to gluconic acid in a minimal amount, suggesting it is a glucose dehydrogenase (Additional file 1: Fig. S7).

As *Ap*AA3_2B, *Ap*AA3_2C, and *Pc*AA3_2A did not oxidize any of the tested substrates, and *Mc*GdhA, *Ab*AA3_2A, and *Ms*AA3_2A were only found to have very limited activity (not quantifiable), these six proteins were omitted from further analysis. The later studies thus focused on six enzymes: *Ps*AaoA (cluster II), *As*AadhA (cluster IV), *As*AadhB (cluster IV), *Ta*GdhA (cluster VI), *Ap*GoxA (cluster XI), and *Ki*OdhA (cluster XXX).

### Kinetic analysis, substrate conversion, and product quantification

#### Aryl alcohol oxidase and dehydrogenases

*Ps*AaoA possesses a very broad substrate specificity. It was able to oxidize all 12 tested aryl alcohols (Table 2). In general, *Ps*AaoA exhibited better catalytic efficiency (*k*_cat_/*K*_M_) and better binding affinity (*K*_M_) with monolignols than with the simple aryl alcohols without the propene group. The best catalytic efficiency (2.2 × 10^5^ M^−1^ s^−1^) and binding affinity (0.05 mM) were observed with cinnamyl alcohol, followed by coniferyl alcohol and *p*-coumaryl alcohol. The best catalytic efficiency and binding affinity towards tested simple aryl alcohols was with *p*-anisyl alcohol (1.0 × 10^4^ M^−1^ s^−1^; 0.47 mM). Consistent with the kinetic analysis, *Ps*AaoA oxidized the cinnamyl alcohol (89%), coniferyl alcohol (84%), and *p*-coumaryl alcohol (74%) to a higher level than *p-*anisyl alcohol (64%) and other simple aryl alcohols after 8 h of incubation (Table 2). The highest turnover rate (*k*_cat_) was recorded on benzyl alcohol (12.4 s^−1^), followed by cinnamyl alcohol (10.5 s^−1^). These aromatic alcohols contain only one substitution on the benzene ring. In contrast, the kinetic parameters could not be measured for 3,5-dimethoxy-4-hydroxybenzyl alcohol and sinapyl alcohol, both of which carry two additional methoxy groups and a hydroxyl group at the benzene ring. Caffeyl alcohol and 5-hydroxymethylfurfural were also found to be oxidized by *Ps*AaoA at around 20%, based on substrate depletion after 8 h incubation (data not shown); however, the kinetic parameters were not measurable. Notably, *p*-anisyl alcohol was clearly preferred over the 4-hydroxybenzyl alcohol, suggesting a preference for the methoxyl group over the hydroxyl group for the substrate binding.

**Table 2:** Kinetic parameters of *Ps*AaoA on aryl alcohols and substrate conversion after 8 h incubation

Substrate	*K*_M_ (mM)	*k*_cat_ (s^−1^)	*k*_cat_/*K*_M_ (M^−1.^s^−1^)	Degree of oxidation (%)
〹 benzyl alcohol	1.6 ± 0.7	12.4 ± 1.5	(7.8 ± 3.7) × 10^3^	29
4-hydroxybenzyl alcohol	9.9 ± 2.0	2.1 ± 0.2	209 ± 47	29
*p*-anisyl alcohol	0.47 ± 0.06	4.7 ± 0.1	(1.0 ± 0.1) × 10^4^	64
4-hydroxy-3-methoxybenzyl alcohol	8.7 ± 1.6	1.7 ± 0.1	200 ± 40	47
3,4-dimethoxybenzyl alcohol	4.0 ± 1.3	1.1 ± 0.1	269 ± 93	56
3,5-dimethoxy-4-hydroxybenzyl alcohol (syringyl alcohol)	n.m.	n.m.	n.m.	3
cinnamyl alcohol	0.05 ± 0.01	10.5 ± 0.4	(2.2 ± 0.4) × 10^5^	89
*p*-coumaryl alcohol	0.15 ± 0.02	3.3 ± 0.02	(2.1 ± 0.3) × 10^4^	74
coniferyl alcohol	0.14 ± 0.01	4.7 ± 0.1	(3.2 ± 0.3) × 10^4^	84
sinapyl alcohol	n.m.	n.m.	n.m.	5

Oxygen was used as electron acceptor, and catalase was added to remove hydrogen peroxide. The substrate conversion was calculated from the product formation

*n.m.* Not measurable

**Fig. 5:**
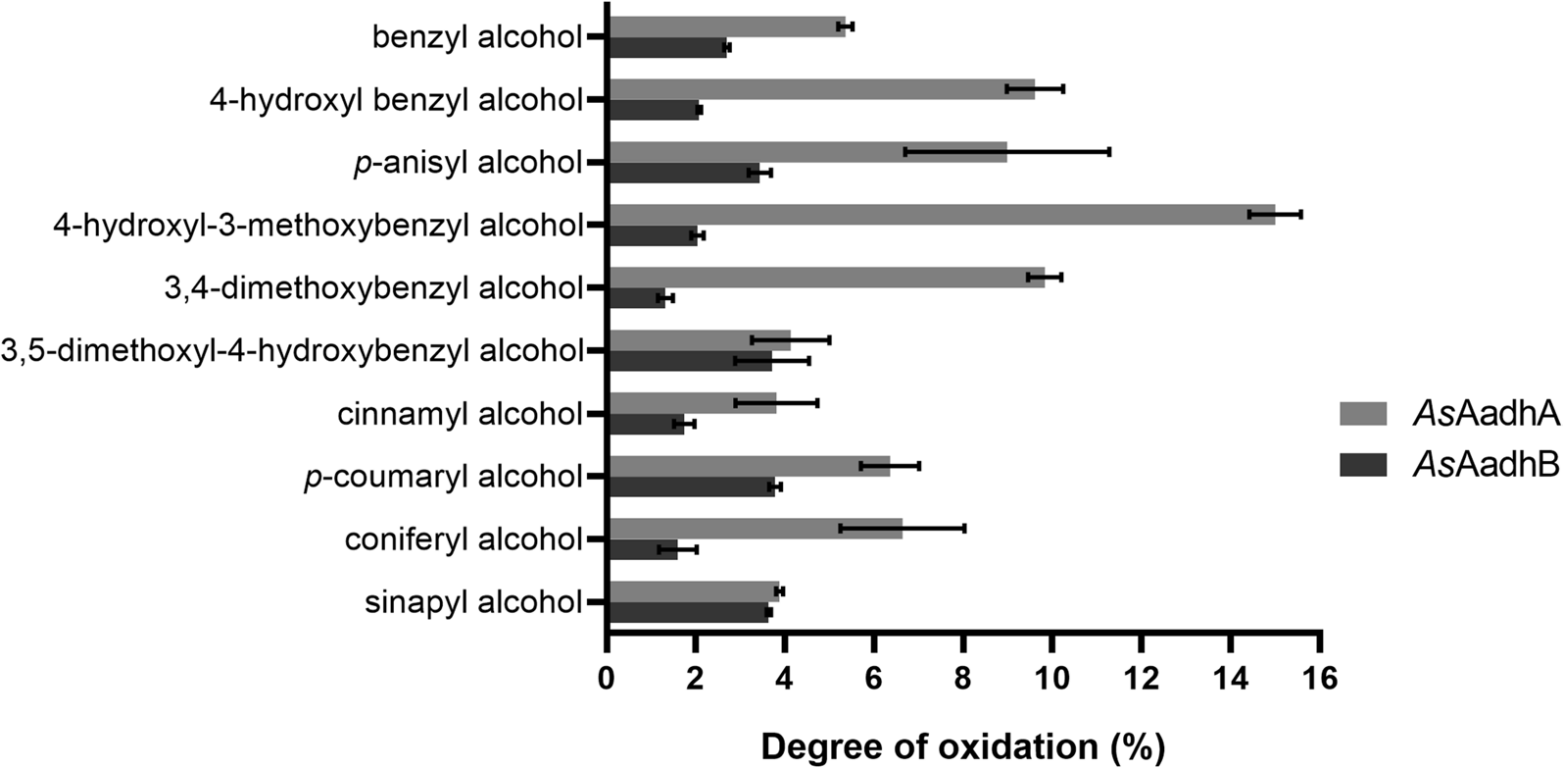
Conversion of aryl alcohols by *As*AadhA and *As*AadhB. The degree of oxidation was calculated from the product formation

The kinetic parameters for *As*AadhA and *As*AadhB with aryl alcohols were not measurable, but they were both found to partly oxidize aryl alcohols during 8 h of incubation (Fig. 5). *As*AadhA showed preference for simple aryl alcohols over monolignols, with the highest conversion detected with 4-hydroxyl-3-methoxybenzyl alcohol, and it was more efficient than *As*AadhB. Interestingly, both *As*AadhA and *As*AadhB depleted 5-hydroxymethylfurfural by about 5% and had no activity on caffeyl alcohol after 8 h of incubation (data not shown).

#### Carbohydrate oxidase and dehydrogenases

Kinetic parameters of three new carbohydrate-oxidizing enzymes were determined with four electron donors: glucose, xylose, mannose, and gentiobiose. *Ap*GoxA displayed the best catalytic efficiency with glucose using molecular oxygen (2.1 × 10^3^ M^−1^ s^−1^) or BQ (1.5 × 10^4^ M^−1^ s^−1^) as the electron acceptor (Table 3). Due to good activity with oxygen, *Ap*GoxA was named a glucose oxidase, although it showed better kinetic efficiency and binding affinity with BQ. While *Ap*GoxA was also able to oxidize gentiobiose, the corresponding kinetic parameters were not measurable due to the low initial velocity and solubility limitation of gentiobiose. *Ta*GdhA was found to oxidize both glucose (3.0 × 10^4^ M^−1^ s^−1^) and xylose (2.0 × 10^4^ M^−1^ s^−1^) with similar catalytic efficiency as when BQ was used as the electron acceptor. Measured *K*_M_ was a bit lower on xylose than glucose. Catalytic efficiency was 20-fold to 30-fold lower on gentiobiose than on glucose, although* K*_M_ on gentiobiose was lower than on glucose (Table 4). *Ki*OdhA was a true oligosaccharide-dehydrogenase, oxidizing gentiobiose with much better catalytic efficiency (3.5 × 10^3^ M^−1^ s^−1^) than with glucose (130 M^−1^ s^−1^). The kinetic parameters for xylose and mannose were not measurable (Table 4). Given the novelty of *Ki*OdhA, further investigations were conducted to assess its ability to deplete monosaccharides and disaccharides in 24-h incubations (Additional file 1: Table S6). Among the tested substrates, the greatest depletion was observed for gentiobiose (78.6%), followed by melibiose (5.1%) and glucose (3.7%).

**Table 3:** Kinetic parameters of *Ap*GoxA with carbohydrate substrates

Substrate	*Ap*GoxA-Oxygen			*Ap*GoxA-BQ		
	*K*_M_ (mM)	*k*_cat_ (s^−1^)	*k*_cat_/*K*_M_ (M^−1.^s^−1^)	*K*_M_ (mM)	*k*_cat_ (s^−1^)	*k*_cat_/*K*_M_ (M^−1.^s^−1^)
Glucose	7.3 ± 0.5	15.5 ± 0.5	(2.1 ± 0.2) × 10^3^	2.9 ± 0.1	41.4 ± 0.8	(1.5 ± 0.6) × 10^4^
Xylose	81.3 ± 4.4	3.1 ± 0.1	38 ± 2	105.5 ± 14.9	3.1 ± 0.2	29 ± 10
Mannose	435.6 ± 23.4	9.2 ± 0.2	21 ± 2	(1.4 ± 0.1) × 10^3^	21.6 ± 0.8	15 ± 10
Gentiobiose	n.m.			n.m.		

Oxygen and BQ were used as electron acceptors. All reactions were conducted at pH 5.5

*n.m.* Not measurable

**Table 4:** Kinetic parameters of *Ta*GdhA and *Ki*OdhA with carbohydrate substrates

Substrate	*Ta*GdhA			*Ki*OdhA		
	*K*_M_ (mM)	*k*_cat_ (s^−1^)	*k*_cat_/*K*_M_ (M^−1.^s^−1^)	*K*_M_ (mM)	*k*_cat_ (s^−1^)	*k*_cat_/*K*_M_ (M^−1.^s^−1^)
Glucose	3.1 ± 0.2	92.5 ± 1.9	(3.0 ± 1.0) × 10^4^	69.0 ± 11.3	9.0 ± 0.8	130 ± 68
Xylose	1.2 ± 0.1	24.3 ± 0.2	(2.0 ± 0.6) × 10^4^	n.m.		
Mannose	524.8 ± 19.2	36.4 ± 0.5	69 ± 25	n.m.		
Gentiobiose	1.7 ± 0.8	1.4 ± 0.2	818 ± 290	3.1 ± 0.7	10.7 ± 1.2	(3.5 ± 1.5) × 10^3^

BQ was used as the electron acceptor. All reactions were conducted at pH 5.5

*n.m.* Not measurable

### AA3_2s can use phenoxy radical as electron acceptor

**Fig. 6:**
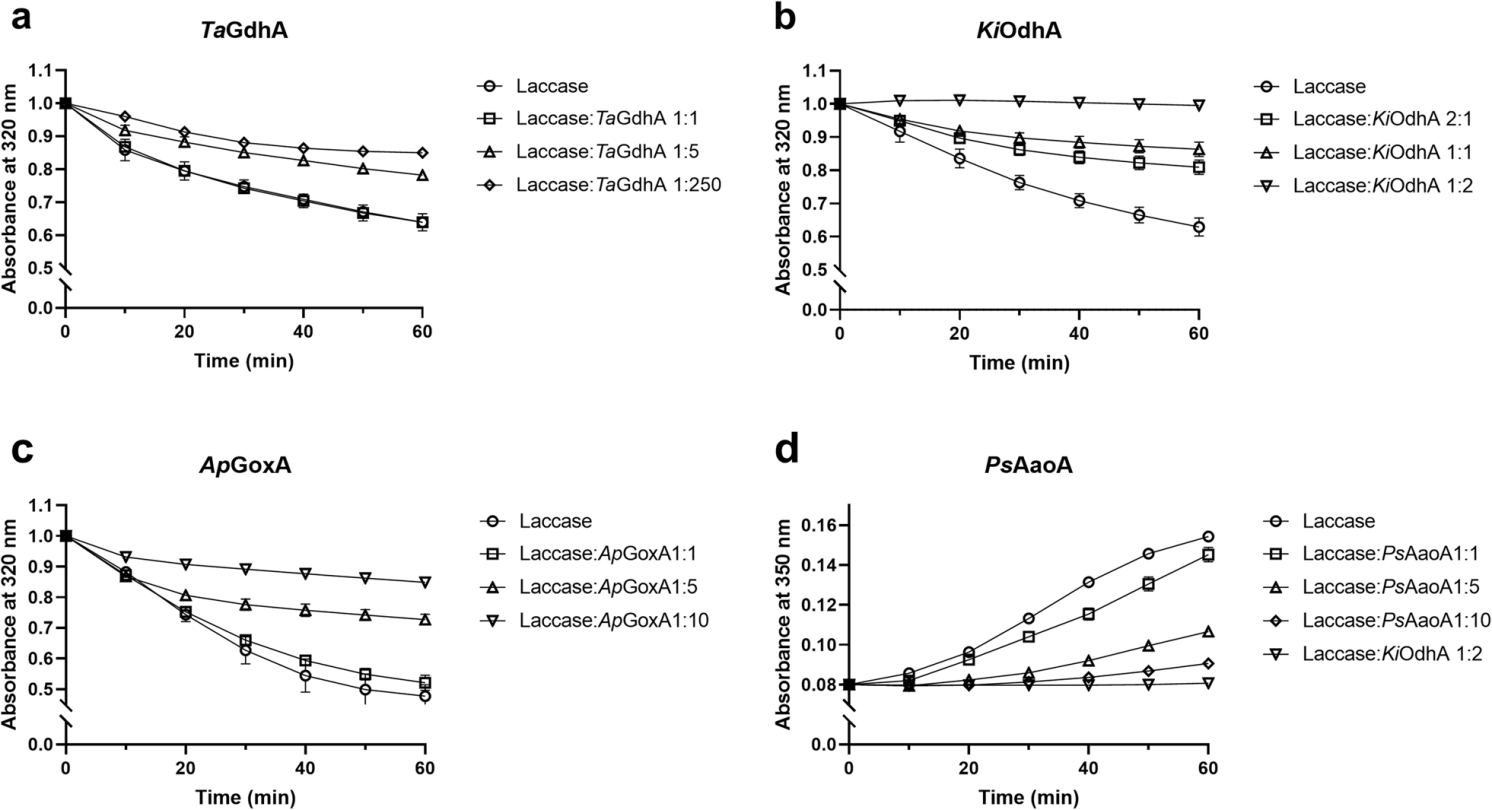
The ability of each AA3_2 to use phenoxy radical as an electron acceptor. Reactions with **a** *Ta*GdhA, **b** *Ki*OdhA, and **c** *Ap*GoxA were followed at 320 nm for the depletion of ferulic acid. **d** *Ps*AaoA was followed at 350 nm for the formation of ferulic acid dimer. Reaction with laccase to *Ki*OdhA in a ratio of 1–2 was also displayed in d for comparison. The enzymes were dosed according to the specific activity of laccase and AA3_2s. For the specific activity of *Ta*GdhA and *Ki*OdhA, BQ was used, and for the specific activity of *Ap*GoxA and *Ps*AaoA, oxygen was used

The AA3_2 enzymes were assessed for their capacity to utilize phenoxy radicals as electron acceptors, which were generated in this study through oxidation of ferulic acid by laccase. In the absence of AA3_2 enzymes, laccase would oxidize ferulic acid to form radicals that subsequently undergo dimerization and polymerization. The consumption of ferulic acid was monitored by measuring the reduction in light absorption at 320 nm for AA3_2s acting on carbohydrates (Fig. 6a–c). *p*-anisyl alcohol, the electron donor for *Ps*AaoA, absorbs light at 320 nm. Due to this interference, we followed ferulic acid dimer formation at 350 nm for *Ps*AaoA (Fig. 6d).

The tested AA3_2 enzymes were observed to facilitate the regeneration of ferulic acid during the laccase oxidation, albeit to varying degrees, by employing the radicals generated by laccase as electron acceptors (Fig. 6). For instance, in the presence of *Ta*GdhA at a 1:5 ratio with laccase after 1 h of reaction, approximately half of the ferulic acid was polymerized (Fig. 6a). A higher enzyme loading ratio of 1:250 with *Ta*GdhA resulted in some improvement in ferulic acid regeneration, although the effect was relatively minor. Conversely, *Ki*OdhA exhibited the most efficient recycling of ferulic acid radicals, achieving a 98.7% recycling rate when used in a laccase-to-*Ki*OdhA ratio of 1:2 after 1 h of reaction (Fig. 6b and Table 5).

**Table 5:** The percentages of remaining ferulic acid after 1 h reaction with/without the addition of catalase in excess amounts

Remaining ferulic acid (%)				
	Laccase:*Ta*GdhA 1:250	Laccase:*Ki*OdhA 1:2	Laccase:*Ap*GoxA 1:10	Laccase:*Ps*AaoA 1:10
control	58.3 ± 0.7	98.7 ± 2.8	71.0 ± 3.9	85.8 ± 0.2
+ catalase	52.7 ± 1.8	97.8 ± 3.1	46.4 ± 4.4	50.7 ± 0.2

Control reactions did not contain catalase

Both oxidases, *Ap*GoxA and *As*AaoA, also seemed to inhibit the dimerization of ferulic acid (Fig. 6c, d). When used at a 1:10 ratio with laccase, *Ap*GoxA and *As*AaoA retained 85.8 and 71.0%, respectively, of the ferulic acid after 1 h of incubation (Table 5). However, under aerobic conditions, oxygen is used by both oxidases as an electron acceptor, which led to the generation of hydrogen peroxide, which is known to inhibit laccase activity [36]. To counteract this inhibitory effect, the reactions were conducted with the addition of an excess amount of catalase. The inclusion of excess catalase had a minimal impact on ferulic acid regeneration for both dehydrogenases (Table 5). On the other hand, with the removal of hydrogen peroxide by catalase in the reactions with *Ap*GoxA and *As*AaoA (in a laccase: AA3_2 ratio of 1:10), the oxidases still demonstrated the ability to use phenoxy radicals as an electron acceptor, with 46.4% and 50.7%, respectively, of the ferulic acid regenerated after 1 h of incubation. This suggests that *Ap*GoxA and *As*AaoA can employ phenoxy radicals as electron acceptors under competition with molecular oxygen.

### Sequence comparison and homology modelling

Multiple sequence alignments were performed on this study’s characterized proteins with some previously characterized AA3_2 enzymes (Additional file 1: Fig. S8). Four regions with amino acid deletions and insertions were identified that might have a role in substrate specificity. To understand the structure–function relationship, the homology models for *Ps*AaoA, *As*AadhA, *As*AadhB, *Ta*GdhA, *Ap*GoxA, and *Ki*OdhA were generated using AlphaFold2 [37]. Most of the residues were modelled with the per-residue confidence (pLDDT) score greater than 90 and the predicted aligned error (PAE) less than 5. The superimposition was then made for the homology models of *Ps*AaoA, *Ta*GdhA, and *Ap*GoxA with the structurally characterized members in the corresponding cluster: *Pe*AAOx from *Pleurotus eryngii* (cluster IIa, PDB_3FIM) [28], *Af*GDH from *Aspergillus flavus* (cluster VI, PDB_4YNT) [30], and *Tc*ODH from *Trametes cinnabarina,* synonym *Pycnoporus cinnabarinus* (previously known as *Pc*GDH; cluster XI, PDB_6XUT) [5], respectively (Additional file 1: Figs. S9–S11). *As*AadhA and *As*AadhB were also aligned with *Pe*AAOx (Additional file 1: Figs. S12 and S13). *Ki*OdhA shared the highest-level sequence identity with *An*GOx from *Aspergillus niger* (39.97%) and *Tc*ODH (38.33%). As both *Ki*OdhA and *Tc*ODH oxidized oligosaccharides, the homology model of *Ki*OdhA was aligned with *Tc*ODH (Additional file 1: Fig. S14).

By the superimposition of the homology models with X-ray structures of previously characterized AA3_2s as well as pair-wise sequence comparison, the key amino acids of the enzymes in the current study were characterized and are summarized in Additional file 1: Fig. S15. To study the conservation of those key amino acids, the sequence logos were made for those key ones within each cluster (Additional file 1: Fig. S16). The significance of variations in these key amino acids for determining the biocatalytic characteristics of the AA3_2 enzymes is addressed in the discussion.

## Discussion

CAZy AA3_2 is a large subfamily that contains enzymes with diverse functionalities. Previous studies of AA3_2s have mainly focused on two clades, namely the PDH-AAO clade and the GDH-GOx clade. These clades, however, account for less than 25% of the current known fungal AA3_2 sequences (Fig. 2a). The characterization of sequences beyond these two clades was limited. To offer a comprehensive view of all the fungal AA3_2 sequences, we employed a SSN which included 4577 fungal AA3_2s from various databases. By annotating the enzymatic functions to the SSN, a clear correlation was seen between enzymatic function and the SSN clustering of AA3_2s (Fig. 1). With the SSN mapping and characterization of new fungal AA3s, enzymatic activities are now discovered from cluster I, II, IV, VI, XI, XII, and XXX, covering over 55% of the total sequence space (Additional file 1: Table S2). We anticipate that this SSN clustering will guide future biochemical characterization of fungal AA3_2s.

The SSN analysis at an e^−420^ cut-off revealed that cluster II corresponds to the previously defined AAO-PDH clade (Figs. 1, 2) [4]. The SSN at an elevated cut-off of e^−470^ divided the PDH sequences to cluster IIb, while the AAO and AAO-like sequences remained together in cluster IIa due to their high level of homology. To the best of our knowledge, no AAO-like protein has been biochemically characterized. Unfortunately, despite the efforts made in this study, *Ab*AA3_2A, which falls in the AAO-like subclade, did not oxidize any of the tested substrates in a way that was readable. Very low levels of activity were detected only on the primary alcohol mixture.

*Ps*AaoA in the SSN cluster II and the AAO subclade in the maximum likelihood tree oxidized a broad spectrum of various aryl alcohols. Similar to previously characterized AAO/AADHs in cluster II, the homology model of *Ps*AaoA carried a conserved hydrophobic tunnel that restricts access to the inner cavity of the active site (Additional file 1: Fig. S9) [25, 28]. In *Pe*AAOx, the hydrophobic tunnel comprised three aromatic residues (Tyr92, Phe397, and Phe501) [28]. The aromatic residue Phe397 is replaced by alanine (Ala427) in *Ps*AaoA. This substitution might contribute to the broader substrate specificity against monolignols by *Ps*AaoA as compared with *Pe*AAOx. *Ps*AaoA demonstrated high levels of catalytic efficiency on cinnamyl alcohol, coniferyl alcohol, and *p*-coumaryl alcohol, whereas *Pe*AAOx showed relatively low catalytic efficiency with cinnamyl alcohol [38]. This is consistent with the broader substrate specificity observed in *Cc*AAO from *Coprinopsis cinerea*, in which the same position is occupied by a non-aromatic amino acid residue, leucine [39].

Two new aryl alcohol dehydrogenases, *As*AadhA and *As*AadhB, were identified in the SSN cluster IV. Interestingly, both *As*AadhA and *As*AadhB carry the CBM1 domain at their N-terminus, which is typically associated with cellulose surface binding [40]. However, it is noteworthy that neither of these enzymes exhibited activity towards the tested carbohydrates. In contrast to the cluster II AAO/AADHs, both *As*AadhA and *As*AadhB lack the three aromatic amino acid residues at the active site, resulting in their fully accessible catalytic sites (Additional file 1: Figs. S12 and S13). This enhanced accessibility appears to be a shared feature among all cluster IV proteins (Additional file 1: Fig. S16), which seems to account for their low levels of oxygen reactivity and catalytic efficiency. This notion finds support from the characterization of another cluster IV enzyme, *Mt*AAOx from *Thermothelomyces thermophilus* (synonym *Myceliophthora thermophila*). *Mt*AAOx also possessed a fully accessible active site, exhibited low catalytic efficiency with tested electron donors, and had low oxygen reactivity [33]. Moreover, mutation of tyrosine to alanine (F501A) in *Pe*AAOx was reported to strongly reduce its reactivity to oxygen [41]. *Ps*AaoA, *As*AadhA, and *As*AadhB showed the ability to oxidize 5-hydroxymethylfurfural (5-HMF), a precursor to 2,5-furandicarboxylic acid, a promising green chemical building block [11, 42]. Nevertheless, their activities with furans are either modest or not comparable to their activities on aromatic alcohols. Further enzymatic engineering work could be considered to enhance their conversion efficiency [43].

The SSN clusters VI, XI, and XII cover the GDH-GOx clade (Fig. 1) [4, 29]. Specifically, cluster XII aligns perfectly with subclade GOx I, which stands out as the most distinct subclade in terms of phylogenetic relationships. This cluster comprises seven characterized glucose oxidases (GOxs) that exhibit a high level of specific activity towards glucose, while xylose is generally a poor substrate for these enzymes [31, 44].

SSN cluster VI corresponds to previously defined subclades GDH I and GDH II. In the present work, two new glucose dehydrogenase, *Ta*GdhA and *Mc*GdhA were identified from SSN cluster VI. *Ta*GdhA, which falls within subclade GDH I, displayed a similar oxidation profile as previously characterized GDHs from GDH I, exhibiting the high levels of catalytic efficiency for glucose oxidation [30, 45, 46]. Notably, while other characterized GDHs from GDH I showed approximately 5–31% activity with xylose compared to glucose, *Ta*GdhA exhibited a higher affinity for xylose (1.2 mM) than glucose (3 mM). The activity of *Mc*GdhA (subclade GDH II) was observed exclusively towards glucose and it was only detectable by sensitive mass spectrometry analysis. This differs from the specificity reported for the subclade GDH II enzyme *Tv*GDH from *Trichoderma virens* that exhibited the highest activity towards maltose, and also oxidized glucose, xylose, and galactose [29]. More biochemical characterization of members in the subclade GDH II would benefit the specificity prediction of enzymes in this subclade.

Subclade GOx II and GDH III are closely related in terms of phylogeny and they fall in SSN cluster XI. Despite their close relationship, GOx II enzymes are believed to be orthologs of GOx I members [4]. Additionally, both subclades, GOx II and GOx I, have a relatively low number of exons compared to GDH subclades and are the only subclades known to exhibit oxygen reactivity [4, 29]. In this study, *Ap*GoxA characterized from the GOx II subclade showed similar substrate specificity to the previously characterized *As*GOxII from *Aureobasidium subglaciale*. Both enzymes had the highest activity towards glucose (Table 3) [29]. Interestingly, *Ap*GoxA and *As*GOxII displayed reactivity towards xylose, which is in contrast to the specific glucose recognition observed in *An*GOx and *Pa*GOx from *Penicillum amagasakiense* within the GOx I subclade [31]. The presence of a threonine or serine residue in *An*GOx (Thr110) and *Pa*GOx (Ser114) was speculated to be responsible for the hydrogen bonding with the C6 of glucose, thus influencing their substrate specificity [31]. However, *Ap*GoxA and *As*GOxII both have serine residues at the same position suggesting that other factors beyond this specific amino acid residue contribute to xylose recognition.

A novel oligosaccharide dehydrogenase *Ki*OdhA was discovered from the SSN cluster XXX. It was found to be phylogenetically closest to GOx I subclade (Fig. 2c). Activity on gentiobiose has also recently been identified from the subclade GDH III members *Tc*ODH, *Um*GDH and *Rs*GDH [5, 24, 29]. However, *Ki*OdhA exhibited remarkable gentiobiose specificity and conversion surpassing previous reports [5, 24, 29]. It displayed at least 15 times higher affinity towards gentiobiose and 5 times higher catalytic efficiency compared to *Um*GDH, making it the most efficient characterized enzyme for gentiobiose oxidation (Table 4) [29]. The recognition and oxidation of oligosaccharides by enzymes have recently gained attention following the re-annotation of *Tc*ODH (*Pc*GDH) [5]. Unlike the hydrogen bonding interactions observed in GDHs or GOxs, *Tc*ODH primarily binds laminaribiose through sugar–aromatic stacking interactions. *Tc*ODH and the homology model of *Ki*OdhA share conserved aromatic amino acid residues (Phe416/Phe435 and Trp430/Trp448) that contribute to the stacking force between the enzyme and the substrate. These residues are highly conserved within SSN clusters XI, XII, and XXX, and the latter residue is also conserved in the cluster VI, suggesting the potential importance of sugar–aromatic stacking interactions in enzymes within these clusters (Additional file 1: Figs. S14 and S16). Intriguingly, Phe421 in *Tc*ODH undergoes a significant shift of approximately 17 Å towards the active site upon binding of laminaribiose, enabling the establishment of sugar–aromatic stacking towards the non-reducing glucosyl moiety [5]. Similar observation was made on *Rs*GDH from *Rhizoctonia solani* and *Um*GDH, both from GDH III, where the same positions are conserved with aromatic residues [29]. However, *Ki*OdhA has a glycine residue at the same position that might explain its low substrate specificity towards laminaribiose. Further structural analysis of *Ki*OdhA would provide valuable insights on its binding mechanism with gentiobiose and substrate specificity.

The activity on disaccharides appears to be a shared characteristic among carbohydrate-oxidizing AA3_2 enzymes from clusters XI, VI, and XXX. In this study, *Ta*GdhA, *Ap*GoxA, and *Ki*OdhA all exhibited activity with disaccharides, particularly gentiobiose with a β−(1 → 6) linkage. Previous research has also shown that enzymes from clusters XI and VI displayed varying degrees of activity with disaccharides such as maltose, laminaribiose, and gentiobiose [5, 29]. Interestingly, *Ki*OdhA is associated with a CBM13 domain at its N-terminus that is typically found in GH16 enzymes, primarily having endo-β-(1 → 3)-glucanase or chitin-β-(1 → 6)-glucanosyltransferase activities [47, 48]. These findings suggest a potential role for AA3_2 enzymes in modifying the fungal cell wall, which contains β-(1 → 3/1 → 6)-linked glucans. This hypothesis is further supported by recent discoveries in a maize pathogen, *Ustilago maydis*, in which the researchers observed co-expression of a glycoside hydrolase specific for β-(1 → 3/1 → 6) glucans and the AA3_2 gentiobiose/laminaribiose oxidase (*Um*GDH) [24]. More studies are needed to reveal the interactions of AA3_2 enzymes and the fungal cell wall to fully understand their physiological role.

This study revealed that both AA3_2 oxidases and dehydrogenases can effectively utilize laccase-generated phenoxy radicals as electron acceptors, in addition to molecular oxygen and quinones. Previous research had already demonstrated that AA3_2 dehydrogenases, including *Tc*ODH, AAQO1-3, and *Gc*GDH, could leverage phenoxy radicals generated by laccase [25, 26, 45]. Our study goes beyond this by showing that phenoxy radicals can compete with molecular oxygen in aerobic environments during the oxidative half-reaction of AA3_2 oxidases, such as *Ap*GoxA and *Ps*AaoA. This remarkable versatile characteristic of AA3_2 enzymes allows them to contribute to lignocellulose degradation through various pathways. For instance, when oxygen is utilized in nature as the electron acceptor, H_2_O_2_ is produced to fuel peroxidase and LPMOs or to initiate the Fenton reaction for the oxidative degradation of lignocellulose [22, 25, 26, 45, 49, 50]. Furthermore, during plant defence events, when cytotoxic quinones are produced, AA3_2 enzymes have the capacity to detoxify them into hydroquinones. These hydroquinones, in turn, can then act as redox mediators to drive LPMOs in the oxidative depolymerization of biomass. Additionally, under conditions of high oxidative stress, AA3_2 enzymes may reduce phenoxy radicals, thereby preventing potential radical cytotoxicity and the repolymerization of semiquinones and other lignin radicals [51]. These results highlight the multifunctionality of AA3_2 enzymes and their potential involvement in multiple stages of lignocellulose degradation.

## Conclusions

The annotation of enzymes in SSN clusters has revealed a significant correlation between SSN clustering and the biochemical functions of these proteins. This provides a foundational framework for the prediction of enzymatic functions of putative AA3_2 sequences. The discovery of three highly active carbohydrate-oxidizing AA3_2s (*Ap*GoxA, *Ta*GdhA, and *Ki*OdhA) and one aryl-alcohol-oxidizing AA3_2 enzyme (*Ps*AaoA) expands the biocatalytic toolbox for future applications in biotechnology and biomedicine.

This study has shed light on the versatility of AA3_2 oxidases and dehydrogenases. Their ability to utilize diverse electron acceptors, especially phenoxy radicals, as an electron acceptor not only mitigates the potential cytotoxicity of radicals but also prevents the re-formation of semiquinones and other lignin radicals. Moreover, the observation of the carbohydrate-active AA3_2 GDH/GOx’s activity, including *Ki*OdhA, on gentiobiose hints at a potential role in modifying fungal cell walls, a biological feature that has thus far been largely overlooked.

## Materials and methods

### Materials

The substrates used in this study, including cellobiose, xylobiose, chitosanbiose, gentiobiose, kojibiose, sophorose, laminaribiose, and nigerose, were purchased from Megazyme Ltd. (Bray, Ireland). The D-glucosamine, D-galactosamine, N-acetyl-D-galactosamine, N-acetyl-D-mannosamine, and D-turanose were obtained from GLYCON Biochemicals GmbH (Luckenwalde, Germany). Sinapaldehyde, 3,5-dimethoxybenzyl alcohol, 4-hydroxycinnamaldehyde, and ferulic acid were purchased from Biosynth Ltd. (Staad, Switzerland). The *p*-coumaryl alcohol was kindly provided by Dr. Anna Happonen. Catalase (C100; Merck & Co., Rahway, NJ, USA) was used in the oxidase reactions to remove hydrogen peroxide, and peroxidase from horseradish (HRP, 77332; Merck & Co., Rahway, NJ, USA) was used in the reactions to couple the hydrogen peroxide formation to 2,2′-azinobis (3-ethylbenzthiazoline-6-sulfonic acid) (ABTS) oxidation. The laccase from *T. versicolor* (38429; Sigma–Aldrich, St. Louis, MO, USA) was used in the reactions to recycle the electron acceptor and to generate radicals. All other buffering chemicals and substrates were purchased from Sigma–Aldrich (St. Louis, MO, USA).

### Phylogenetic analysis and generation of the sequence similarity network (SSN)

AA3_2 proteins used in this study were collected from three fungal sequence resources: MycoCosm (https://mycocosm.jgi.doe.gov/mycocosm/home), MycoCLAP [34] and all 33 fungal genomes from CSFG at Concordia University. Protein sequences predicted from all published fungal genomes in MycoCosm were downloaded in June 2018. Proteins from MycoCLAP and CSFG were collected in May 2019. To determine proteins containing the AA3_2 domain, detection was performed using hmmscan from the HMMER package (http://hmmer.org/) and hidden Markov models (HMMs) from the dbCAN version 6.0 database [52]. The HMMs of GH74 and CE1 enzyme families from dbCAN were replaced by in-house HMMs, to increase the accuracy of the search and to detect potential overlap between domains. In addition, for CBM10, the HMM from the Pfam database [53] was used. The search result gained seven biochemically characterized AA3_2 proteins from MycoCLAP, 4450 AA3_2s from MycoCosm, and 304 AA3_2s from CSFG. AA3_2 domain regions were trimmed from the whole protein sequences by in-house Perl scripts. The final dataset, after removing duplicates, consists of 4577 AA3_2 protein sequences and 4578 AA3_2 domain sequences.

The final dataset for the phylogenetic analysis of fungal AA3_2 sequences consisted of sequences obtained from the database search in addition to sequences annotated biochemically from previous publications. Redundant sequences with 100% identity were consolidated into a single sequence (Additional file 2). The signal peptides and other additional modules were trimmed out, leaving the GMC domain for subsequent phylogenetic analysis (Additional file 3). An SSN was generated using the SSNpipe [47], and the edge threshold cut-off was adjusted using bit scores between 400 and 500 in steps of 10. The SSN was visualized and edited by cystoscape using the yFiles organic layout [54]. The subgroup of each fungal sequence is listed in Additional file 4. The nodes representing the characterized proteins were then marked by color according to their enzymatic specificities. The domain sequences comprising more than 370 amino acids were aligned using MAFFT v.7.505 using the default parameters on CIPRES Science Gateway, and the informative sites were selected using ClipKit’s default settings [55–57]. The domain sequences were also sorted based on their clustering in the SSN, and the sequences in clusters IIa, IIb, IV, VI, XI, XII, XXX were also aligned individually using MAFFT, with the informative sites selected by ClipKit. The conservation of the key amino acids in each cluster was visualized with WebLogo [58].

With the multiple sequence alignments, three maximum likelihood phylogenetic trees were generated. One tree consists of all the aligned AA3_2 sequences, another consists of the sequences within cluster II, and the third tree consists of the sequences within clusters VI, XI, XII, XVIII, XXIII, XXIV, and XXX. The trees were generated using RAxML v.8 with 100 bootstrap replications on the CIPRES Science Gateway portal [59]. The resulting trees were rooted at midpoints visualized in FigTree (Newick files of the trees in Additional file 5). The clades in the trees were defined based on the bootstrap values, topology, characterized sequences, and the old tree generated by [4]. The homology models of *Ps*AaoA, *As*AadhA, *As*AadhB, *Ta*GdhA, *Ki*OdhA, and *Ap*GoxA were generated using the CoLab version of AlphaFold2 and visualized with PyMOL V2.0 [37].

### AA3_2 genes and recombinant protein production

Based on the phylogenetic analysis, 27 AA3_2 genes distributed in different clusters were then selected for production (Additional file 1: Table S3). To express and secrete AA3_2 proteins into the medium of *Pichia pastoris*, the coding sequences of 27 *aa*3_2 genes without the sequences of signal peptide and stop codon were cloned into the plasmid pPICZα A. The *aa*3_2 sequences of interest were amplified using PCR by Phusion DNA Polymerase (New England Biolabs, Ipswich, MA, USA) and the cloning plasmids pJETC carrying *aa*3_2 genes as the templates. The primers used were designed with Geneious software. Forward primers of 27 *aa*3_2 genes anchored the sequences of *Xho* I recognition site and Kex2 signal cleavage, while reverse primers were added to the sequences of 6xHis_tag, stop codon, and *Xba* I recognition sites. The melting temperatures (*T*_*m*_) of the primers were calculated to range from 55 to 67 ℃, and primer pairs had *T*_*m*_ within 5 ℃ of each other. The PCR products and the *Xho* I_*Xba* I digestion reactions of PCR products were purified using the EZ-10 Spin Column PCR Products Purification Kit (Bio Basic Inc., Markham, Ontario, Canada), while the *Xho* I_*Xba* I reactions of plasmid pPICZα A were purified through agarose gel and extracted using the EZ-10 Spin Column DNA Gel Extraction Kit (Bio Basic Inc., Markham, Ontario, Canada). The ligation reactions of *Xho* I_*Xba* I-treated PCR products and *Xho* I_*Xba* I-treated pPICZα A were carried out overnight at 16 ℃ using T4 DNA Ligase (New England Biolabs, Ipswich, MA, USA). The overnight ligation mixtures were chemically transformed into *E. coli* DH5α, spread onto LB agar medium plates containing 25 µg/mL Zeocin antibiotics (InvivoGen, San Diego, CA, USA) [1], and incubated overnight at 37 ℃. Expected recombinant plasmids in the Zeocin-resistant *E. coli* colonies were extracted and verified using *Xho* I and *Xba* I digestions and analysis of molecular weights on agarose gel.

The selected recombinant plasmids pPICZα A-*aa*3_2s were completely linearized using the restriction enzyme *Pme* I or *Sac* I and purified using the EZ-10 Spin Column PCR Products Purification Kit (Bio Basic Inc., Markham, Ontario, Canada). Competent *P. pastoris* KM71H cells were electroporated with 2–5 µg of purified linearized recombinant plasmids. After pulsing, 1 mL of ice-cold 1 M sorbitol was immediately added to the electroporation cuvette to re-suspend *P. pastoris* KM71H cells. Then, the cuvette contents were transferred to a sterile 15-mL Falcon tube and incubated at 30 ℃ without shaking for 2 h. The electroporated *P. pastoris* KM71H cells were spread on YPDS agar plates containing 100 µg/mL Zeocin using 100–250 µL of the cuvette contents and incubated at 30 ℃ for 3 days to obtain colonies. Five *P. pastoris* colonies of each clone were selected to screen for the expression of AA3_2 proteins. Some cells of each *P. pastoris* colony were transferred into 2 mL of YPD broth containing 100 µg/mL Zeocin using a sterile toothpick and shaken at 200 rpm and 30 ℃ for 20–22 h. Then, around 200 µL of overnight cultures were transferred into 5 mL of buffered glycerol complex medium (BMGY) and shaken at 250–300 rpm and 30 ℃ for 22–24 h. The cells of *P. pastoris* were harvested using the centrifugation at 2000 g and 18 ℃ for 5 min, then re-suspended by adding 2 mL of buffered methanol complex medium (BMMY) and shaken at 250–300 rpm and 20 ℃ for 72 h. Methanol was added every 20–24 h to a final concentration of 1% to maintain induction. The supernatants of 72 h shaken *P. pastoris* KM71H cultures were collected by centrifuging at 2500 g and 4 ℃ for 10 min and then stored at 4 ℃ for analysis the next day or at −80 ℃ for long-term storage. The expression levels of recombinant AA3_2s of the *P. pastoris* KM71H colonies were analyzed with Coomassie-stained SDS-PAGE using 10 µL of supernatant to load on gel.

Successful transformants were used to produce recombinant proteins. First, a preculture was grown using 10 mL of BMGY inoculated from a YPDZ plate, then shaken in a 50 mL Falcon tube at 200 rpm and 30 ℃ until an OD600 of 5.5–6.5 was reached. Then, 1 L of BMGY in a 4 L shake flask was inoculated with 2 mL of the preculture. This BMGY culture was left shaking at 200 rpm and 30 ℃ overnight to grow to sufficient cell density. The next day, the culture was pelleted at 3000 g and re-suspended in 200 mL of BMMY with 1% methanol to promote protein expression. Then, the culture was shaken at 200 rpm and 15 °C. For the next 3 days, 2 mL of methanol was added each day to replenish consumed methanol. After the third day of methanol consumption, cells were pelleted, and the supernatant was filtered using 0.45 μm capsule filters (Cytiva Whatman Polycap TC; Cytiva Life Sciences, Marlborough, MA, USA) to prepare for purification. To reach adequate protein yields, 5–10 BMGY cultures for each protein were grown at the same time, and BMMY cultures were pooled to make 1 L total volumes. Protein yield was measured by SDS-PAGE to ensure adequate expression before purification.

### Purification of the recombinant protein

The secreted recombinant proteins were first concentrated to a smaller volume using a centrifuge filter with a cut-off of 10 kDa. Afterwards, the concentrated fraction was filtrated through a 0.45 µm filter and loaded to HisTrap HP columns (GE HealthCare, Chicago, IL, USA) in buffer A (50 mM Tris–HCl buffer at pH 7.8 with 0.15 M sodium chloride and 10 mM imidazole). The impurities were subsequently removed with a 10-column volume of buffer A. The tagged proteins were then eluted with 50% of buffer B (50 mM Tris–HCl buffer at pH 7.8 with 0.15 M sodium chloride and 10 mM imidazole). The elution was tracked by UV absorption at 280 nm, and the fractions containing the tagged protein were pooled together, concentrated, and buffer exchanged to 10 mM Na acetate at pH 5 using 30 kDa cut-off Vivaspin 20 spin columns (Sartorius, Göttingen, Germany). The proteins were run with SDS-PAGE and the final protein concentration was determined using a bicinchoninic acid assay (BCA; Thermo Fisher, Waltham, MA, USA). The purified protein was snap-frozen and stored at −80 ℃ in aliquots.

The presence of oxidized FAD in the purified protein was checked by scanning the purified protein (around 10 mg/ml) with a spectrophotometer (Shimadzu UV-2501; Shimadzu Corporation, Kyoto, Japan) from 320 to 550 nm. *Ap*AA3_2C (FAD missing) was loaded with FAD by incubating the protein with 50-fold excess free FAD overnight in darkness. All later reactions were performed with the FAD-containing proteins and *Ap*AA3_2C with external FAD.

### Enzymatic assays

#### Preliminary activity screening

The dehydrogenase activity of an AA3_2 protein was assayed using the two most commonly used electron acceptors, BQ and DCIP, while the oxidase activity was screened by coupling the H_2_O_2_ formation to the oxidation of ABTS by horseradish peroxidase (ABTS-HRP). The enzyme activity was screened in 50 mM sodium acetate buffer at pH 5 on 14 different substrate mixtures, including 52 individual substrates (Additional file 1: Table S4). The selection of substrates was made to achieve the greatest level of structural diversity. Reactions were performed in 96-well plates in triplicate, and each reaction was loaded with 0.02 µg/µl purified AA3_2 protein and the corresponding e-acceptor (1 mM BQ, 0.2 mM DCIP, or 2 mM ABTS combined with 1.5 units of HRP). The reactions were monitored with an Eon plate reader for 1 h at 40 s intervals for the first 15 min, and 3 min intervals for the following 45 min at 30 ℃. After 24 h at room temperature and in the dark, the light absorbance was again measured. The initial activity was followed according to the corresponding electron acceptor, BQ (ε_abs290_ = 2.24 mM^−1^ cm^−1^), DCIP (ε_abs520_ = 7.8 mM^−1^ cm^−1^), and ABTS (ε_abs420_ = 3.6 mM^−1^ cm^−1^), apart from the dehydrogenase activity assay on aryl alcohols with BQ due to an interference in light absorption. Therefore, the dehydrogenase activity test with BQ on aryl alcohol mixture 1 was monitored semi-quantitatively at 290 nm, while the dehydrogenase activity test with BQ on aryl alcohol mixture 2 and monolignol mixture was monitored semi-quantitatively at 320 nm (details in Additional file 1: Table S7). For the BQ and DCIP reactions, the oxygen concentration was not regulated.

#### Secondary activity screen

The substrates contained within the oxidized mixtures were then screened individually with the corresponding protein and electron acceptor. Taking *As*AadhA as an example, *As*AadhA oxidized the aryl alcohol mixtures and monolignol mixture using BQ as an electron acceptor. Therefore, in the second level of screening, the activity of *As*AadhA was evaluated against the individual substrates within those mixtures with the presence of BQ. The reaction conditions were identical to those stated in the preliminary activity screening section, with the changes in the wavelengths to be followed and the extinction coefficient to be employed (details in Additional file 1: Table S7).

#### pH optimum and kinetic measurements

Using the best accepted electron acceptor and substrate, the pH profiles were determined for the proteins with at least modest activity detected in the screening (*Ps*AaoA, *Ap*GoxA, *As*AadhA, *As*AadhB, *Ap*AA3_2B, *Ta*GdhA, *Ki*OdhA). The pH optimum for *Ps*AaoA as an oxidase was assayed with 1 mM of cinnamyl alcohol (ε_abs290_ = 16.21 mM^−1^ cm^−1^) in the absence of HRP and ABTS. In contrast, the oxidase activity of *Ap*GoxA at various pH levels was evaluated using 10 mM of glucose and the presence of HRP and ABTS (ε_abs420_ = 3.6 mM^−1^ cm^−1^). All dehydrogenase activities were measured using BQ as the electron acceptor, 10 mM glucose as the electron donor for *Ap*AA3_2B and *Ta*GdhA, 10 mM gentiobiose for *Ki*OdhA, and 10 mM 4-hydroxylbenzyl alcohol (ε_abs290_ = 18.24 mM^−1^ cm^−1^) for *As*AadhA and *As*AadhB. All pH optimum assays were conducted in triplicate at 30 ℃, pH values from 3.0 to 8.0, using 100 mM sodium citrate and sodium phosphate buffer. Steady-state kinetic constants were measured using the optimum pH for each protein with the corresponding electron acceptor in triplicate. The reduction rate of the e-acceptor or the formation of the oxidized e-donor was plotted versus substrate concentration (8 points minimum). The Michaelis–Menten constant (K_m_) was estimated by fitting the data to the Michaelis–Menten equation using GraphPad Prism 6.0 (GraphPad Software, La Jolla, CA, USA). Laccase activity was measured using 5 mM hydroquinone (HQ, H9003; Sigma–Aldrich, St. Louis, MO, USA), and oxidation of HQ (ε_abs249_ = 17.25 mM^−1^ cm^−1^) in 250 μl reaction was followed at 249 nm.

### Product analysis with ESI-Q-ToF–MS, HPAEC-PAD, and UPLC-PDA

*ESI-Q-ToF–MS for identifying the oxidized carbohydrates.* The study was performed on the reaction mixture comprising 0.02 µg/µl of each carbohydrate-oxidizing protein, 10 mM glucose (for *Ap*GoxA and *Ta*GdhA) or 10 mM gentiobiose (for *Ki*OdhA) as substrate, and 10 mM sodium acetate buffer at pH 5.5. Catalase (0.5 U/ml) was added in the reactions containing *Ap*GoxA to eliminate H_2_O_2_. BQ (0.2 mM) was incorporated into the dehydrogenase reactions as an electron acceptor and *T. versicolor* laccase (0.5 U/ml) was added to regenerate the BQ. With *Pc*AA3_2A and *Mc*GdhA (0.02 µg/µl each), they were reacted with 10 mM glucose, xylose, mannose, galactose, and L-arabinose in pH 5.5 10 mM sodium acetate buffer. *Ap*AA3_2B was put to reaction with 10 mM glucose, xylose, galactose, nigerose, maltose, isomaltose, sophorose, and cellobiose. All reactions were stopped after 24 h of incubation at 30 ℃ with shaking (400 rpm) and then filtered through a 10 kDa centrifuge filter. Mass spectrometric analysis was conducted to identify the carbohydrate-based reaction products using quadrupole time-of-flight (Q-ToF) coupled with an ESI source (SYNAPT G2-Si; Waters Corporation, Milford, MA, USA). The samples were then dissolved in 50% methanol containing 0.1 mg/ml ammonium chloride with a substrate concentration of 0.1 mg/ml. The analysis was conducted in negative mode, and the ions were collected in a *m/z* range of 50–600 with the parameters developed by [10].

#### Quantification of carbohydrate depletion by HPAEC-PAD

High-performance anion-exchange chromatography coupled with pulsed amperometric detection (HPAEC-PAD) was then used to quantify the substrate depletion by *Ki*OdhA. Reactions were performed at 30 ℃ for 24 h with shaking (400 rpm) in 10 mM ammonium acetate buffer at pH 5.5 containing 5 mM of substrates (Table 1). *Ki*OdhA was dosed at 0.001 µg/µl, and the tested substrates included gentiobiose, glucose, xylose, mannose, L-arabinose, isomaltose, laminaribiose, and melibiose. BQ (0.2 mM) was incorporated into the reactions as an electron acceptor, and *T. versicolor* laccase (0.5 U/ml) was added to regenerate the BQ. All reactions were performed in duplicate and were stopped by filtering through a 10 kDa centrifuge filter. A 4 × 250 mm CarboPac PA-1 column with CarboPac guard column (Dionex Corporation, Sunnyvale, CA, USA) was used for the separation. The elution reagents utilized were (A) water, (B) 100 mM NaOH, and (C) 1 M sodium acetate in 100 mM NaOH. The samples were eluted at 1 ml/min with an isocratic elution of 4 mM NaOH for the first 20 min, then the concentration of NaOH was increased from 20 to 30 min, reaching 100 mM at 30 min. From 30 to 55 min, the concentration of NaOH was kept constant. However, the concentration of sodium acetate increased from 0 to 120 mM between 30 and 45 min, from 120 to 200 mM between 45 and 50 min, and from 200 mM back to 0 mM between 50 and 55 min. From 55 to 60 min, the NaOH concentration returned to 4% and was maintained at that level for an additional 5 min. The detector was in the pulse mode at 30 ℃. The pulse potentials and durations were E1 = 0.05 V, T1 = 400 ms, E2 = 0.75 0 V, t2 = 120 ms, E3 = −0.8 V, t3 = 130 ms, and ts = 20 ms.

#### UPLC-PDA for the oxidation of aryl alcohols

The aryl alcohol oxidase/dehydrogenase reactions were performed with 1 mM of substrate under shaking (400 rpm) in 10 mM sodium acetate buffer (pH 5.0). Catalase (0.5 U/ml) was added in the reactions containing *Ps*AaoA (0.0029 µg/µl) to eliminate H_2_O_2_. BQ (2 mM) was incorporated into *As*AadhA and *As*AadhB reactions as an electron acceptor. The *Ps*AaoA loading was optimized so that the 3,4-dimethoxy-4-hydroxybenzyl alcohol was fully converted after 8 h of reaction; 0.02 µg/µl *As*AadhA and *As*AadhB was loaded in the reactions. All of the reactions were performed in duplicate and in the dark at 30 ℃ for 8 h. The termination of the reactions was achieved by filtering through a 10 kDa centrifuge filter. The reaction product identification and quantification were conducted using an Acquity UPLC coupled with photodiode array (PDA) detector. The substrates and reaction products were separated using an Acquity UPLC HSS PFP column (100 Å, 1.8 µm, 2.1 mm × 100 mm; Waters Corporation, Milford, MA, USA). The mobile phases were A) 0.1% formic acid in acetonitrile and B) 0.1% formic acid in water. The elution gradient was as follows: from 95% A to 80% A in 4 min, isocratic (80% A) for 2 min, then from 80% A to 55% A in 6 min, an immediate change to 5% A and isocratic at 5% A for 2 min, and finally, back to 95% A for a 2 min re-equilibrium to the initial condition. The flow rate was 400 µl/min and the column temperature was set at 40 ℃. The PDA settings were a wavelength range of 210–550 nm, 5 data points/s, and resolution of 1.2 nm. The external standard series were produced by injection from 0.025 nmol to 6 nmol of the aryl alcohols and their corresponding aldehydes. The conversions of caffeyl alcohol and 5-hydroxymethylfurfural were quantified with substrate depletion due to the lack of aldehyde standards, while all other conversions were quantified by product formation with corresponding aldehyde standards. The injection volume for each sample was adjusted so that the amount of each substrate and its reaction product fell within the quantification range. All the reactions were carried out in duplicate.

### Laccase-generated phenoxy radical recycling assays

The assays were performed at pH 5.5 and 30 ℃ in 20 mM ammonium acetate with 0.1 mM ferulic acid, laccase from *T. versicolor* at a concentration of 40 U/L, and 5 mM glucose or 5 mM anisyl alcohol. *Ta*GdhA and *Ki*OdhA were dosed at different concentrations from 20 U/L to 10,000 U/L. Negative controls were performed with the presence of boiled AA3_2 dehydrogenases. Catalase at a concentration of 2 U/ml was included in some reactions as a control to eliminate hydrogen peroxide. The remaining ferulic acid was followed by spectrophotometer at 320 nm, and the formation of ferulic acid dimer was followed by spectrophotometer at 350 nm for 60 min with 10 min intervals. Averages and standard deviations were calculated over three replicate reactions (*n* = 3).

### Supplementary Information

**Additional file 1: Table S1.** List of the previously biochemically characterized proteins with the information of the database source, source organism, strain, protein activity, name, and the related publication. **Table S2.** Statistics of the major SSN clusters. **Table S3.** The AA3_2 sequences that were selected in this study with the database source, organism, and the production status and the biochemical information. **Table S4.** The list of substrates that were tested for the activity assays. **Table S5.** UPLC PDA retention time for each compound and the spectrum of each compound. **Table S6.** Substrate depletion by KiOdhA followed by HPAEC-PAD after 24 h incubation. **Table S7.** The extinction coefficient and wavelength to be used for the activity assay on aryl alcohols under different pH. **Fig S1.** SSN at the cut-off of 470 for the further division of cluster II. **Fig S2.** Absorption spectra of the concentrated AA3_2s. The oxidized FAD should have two absorbance maxima at 375-380 nm and at 440-444 nm. **Fig S3.** SDS page gel of the successfully produced AA3_2 proteins. **Fig S4.** UPLC-PDA Chromatogram (290 nm) of a) Standards of coniferyl alcohol, coniferaldehyde, ferulic acid, benzoquinone and hydroquinone b) Coniferyl alcohol after 8 h incubation with boiled PsAaoA at 30 °C (C) Coniferyl alcohol after PsAaoA oxidation for 8 h at 30 °C, showing the formation of coniferaldehyde. **Fig S5.** Mass spectra collected in negative ion mode showing a) Glucose b) Glucose after oxidation by ApGoxA and c) Glucose after oxidation by TaGdhA. **Fig S6.** Mass spectra collected in negative ion mode showing a) Gentiobiose b) Gentiobiose after oxidation by KiOdhA. **Fig S7.** Mass spectra collected in negative ion mode showing a) Glucose; b) Glucose after incubation with ApAA3_2B; c) Glucose after incubation with PcAA3_2A; and d) Glucose after incubation with McGdhA. **Fig S8.** Multiple Sequence Alignment (MSA) of characterized AA3_2 members in this study and previously. Red boxes show the primary sequence differences between the different enzymes. **Fig S9.** a) Surface and b) ribbon and sticks (active site and FAD) of the AlphaFold homology model of PsAaoA. The FAD and catalytic residues colored in green, hydrophobic residues to form the tunnel to block free access to active site are shown in orange, and the unique motifs identified from MSA are shown in Cyan. c) Alignment for the active site of PsAaoA (Red) and PeAAOx (white, PDB: 3FIM). **Fig S10.** a) Surface and b) ribbon and sticks (active site and FAD) of the AlphaFold homology model of TaGdhA. The FAD and catalytic residues colored in green, residues for substrate binding are shown in orange, and the unique motifs identified from MSA are shown in cyan. c) Alignment for the active site of TaGdhA (blue), AfGDH (white, PDB: 4YNT), and AfGDH in complex with D-glucono-1,5-lactone (pink, PDB: 4YNU). **Fig S11.** a) Surface and b) ribbon and sticks (active site and FAD) of the AlphaFold homology model of ApGoxA. The FAD and catalytic residues colored in green, residues for substrate binding are shown in orange, and the unique motifs identified from MSA are shown in cyan. c) Alignment for the active site of ApGoxA (blue), TcODH (white, PDB: 6XUT), and TcODH in complex with glucose (pink, PDB: 6XUU). **Fig S12.** a) Surface and b) ribbon and sticks (active site and FAD) of the AlphaFold homology model of AsAadhA. The FAD and catalytic residues colored in green and the unique motifs identified from MSA are shown in cyan. c) Alignment for the active site of AsAadhA (Red) and PeAAO (white, PDB: 3FIM). **Fig S13.** a) Surface and b) ribbon and sticks (active site and FAD) of the AlphaFold homology model of AsAadhB. The FAD and catalytic residues colored in green and the unique motifs identified from MSA are shown in cyan. c) Alignment for the active site of AsAadhB (Red) and PeAAO (white, PDB: 3FIM). **Fig S14.** a) Surface and b) ribbon and sticks (active site and FAD) of the AlphaFold homology model of KiOdhA. The FAD and catalytic residues colored in green, residues for substrate binding are shown in orange, and the unique motifs identified from MSA are shown in cyan. c) Alignment for the active site of KiOdhA (blue), TcODH (white, PDB: 6XUT), and TcODH in complex with glucose (pink, PDB: 6XUU). **Fig S15.** Amino acids and positions within the characterized AA3_2 sequences that are implicated in catalysis and substrate preference. **Fig S16.** Sequence logos of the active site residues from clades IIa, IIb, IV, VI, XI, XII, and XXX. The amino acid numbering of the sequences is based on PsAaoA for cluster IIa, AmPDH1 for cluster IIb, AsAadhA for cluster IV, TaGdhA for cluster VI, ApGoxA for cluster XI, AnGOx for cluster XII, and KiOdhA for cluster XXX.

**Additional file 2:** The fasta file of all fungal AA3_2 sequences in full length.

**Additional file 3:** The fasta file of all fungal AA3_2 domain sequences.

**Additional file 4:** The txt file containing the cluster information of all fungal AA3_2 sequences.

**Additional file 5:** The maximum likelihood trees of all AA3_2s, AAO/PDH clade, and GDH/GOx clade in Newick format.

## Data Availability

The datasets generated for this study are available on request to the corresponding author.
