# Supplementary material for: Substrate specificity mapping of fungal CAZy AA3_2 oxidoreductases
Source: Biotechnol Biofuels Bioprod. 2024 Mar 27;17:47. doi: 10.1186/s13068-024-02491-8 (PMC10967070; doi:10.1186/s13068-024-02491-8)
Supplement: Supplementary file 1 — Additional file 1: Table S1. List of the previously biochemically characterized proteins with the information of the database source, source organism, strain, protein activity, name, and the related publication. Table S2. Statistics of the major SSN clusters. Table S3. The AA3_2 sequences that were selected in this study with the database source, organism, and the production status and the biochemical information. Table S4. The list of substrates that were tested for the activity assays. Table S5. UPLC PDA retention time for each compound and the spectrum of each compound. Table S6. Substrate depletion by KiOdhA followed by HPAEC-PAD after 24 h incubation. Table S7. The extinction coefficient and wavelength to be used for the activity assay on aryl alcohols under different pH. Fig S1. SSN at the cut-off of 470 for the further division of cluster II. Fig S2. Absorption spectra of the concentrated AA3_2s. The oxidized FAD should have two absorbance maxima at 375-380 nm and at 440-444 nm. Fig S3. SDS page gel of the successfully produced AA3_2 proteins. Fig S4. UPLC-PDA Chromatogram (290 nm) of a) Standards of coniferyl alcohol, coniferaldehyde, ferulic acid, benzoquinone and hydroquinone b) Coniferyl alcohol after 8 h incubation with boiled PsAaoA at 30 °C (C) Coniferyl alcohol after PsAaoA oxidation for 8 h at 30 °C, showing the formation of coniferaldehyde. Fig S5. Mass spectra collected in negative ion mode showing a) Glucose b) Glucose after oxidation by ApGoxA and c) Glucose after oxidation by TaGdhA. Fig S6. Mass spectra collected in negative ion mode showing a) Gentiobiose b) Gentiobiose after oxidation by KiOdhA. Fig S7. Mass spectra collected in negative ion mode showing a) Glucose; b) Glucose after incubation with ApAA3_2B; c) Glucose after incubation with PcAA3_2A; and d) Glucose after incubation with McGdhA. Fig S8. Multiple Sequence Alignment (MSA) of characterized AA3_2 members in this study and previously. Red boxes show the primary sequence differences [file 13068_2024_2491_MOESM1_ESM.docx]

**Additional File 1**

Substrate specificity mapping of fungal CAZy AA3_2 oxidoreductases

Hongbo Zhao^1^, Johanna Karppi^1,4^, Owen Mototsune^2^, Daria Poshina^1^, Jenny Svartström^1^, Thi Truc Minh Nguyen^3^, Tri Minh Vo^3^, Adrian Tsang^3^, Emma Master^2,4^, Maija Tenkanen^1^*

*Correspondence: hongbo.zhao@helsinki.fi

^1^ Department of Food and Nutrition, University of Helsinki, Helsinki, Finland

^2^ Department of Chemical Engineering and Applied Chemistry, University of Toronto, Toronto, ON, Canada

^3^ Centre for Structural and Functional Genomics, Concordia University, 7141 Sherbrooke Street West, Montreal, QC, H4B 1R6, Canada

^4^ Department of Bioproducts and Biosystems, Aalto University, Espoo, Finland

**
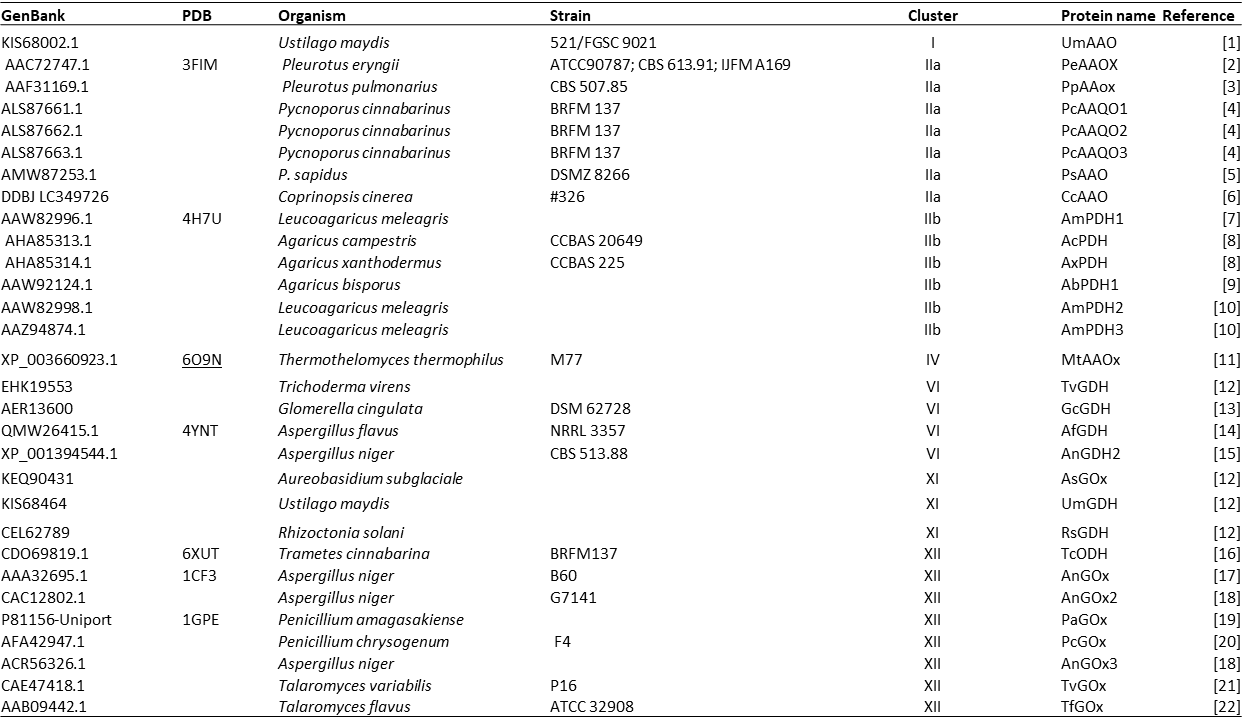
**Table S1. List of the previously biochemically characterized proteins with the information of the database source, source organism, strain, name, and the related publication.

Table S2. Statistics of the major SSN clusters.


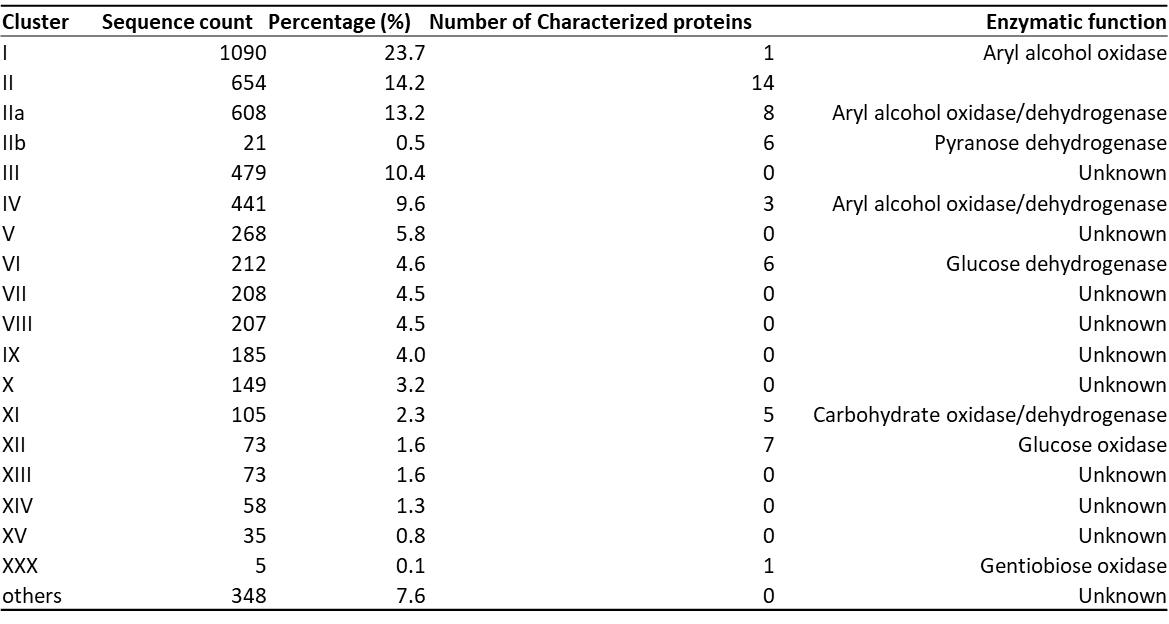


**
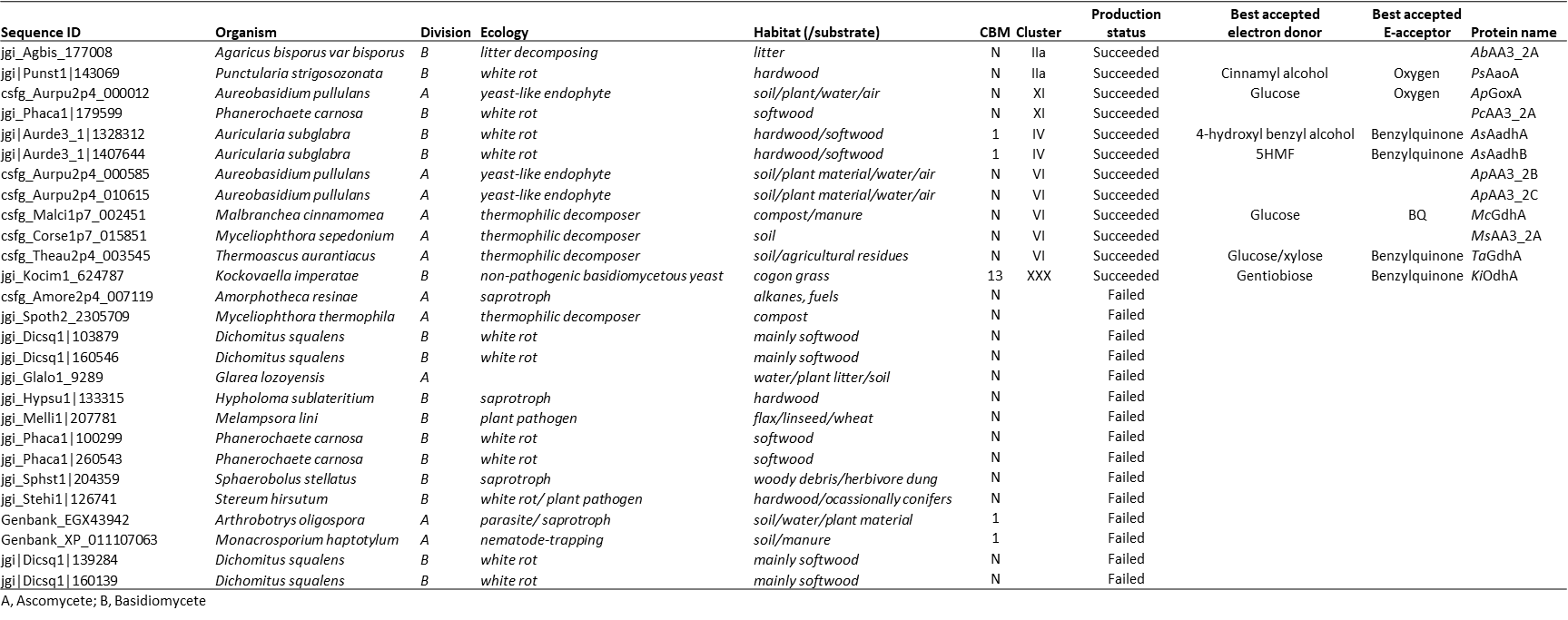
**Table S3. The AA3_2 sequences that were selected in this study with the database source, organism, production status, and biochemical information.

**
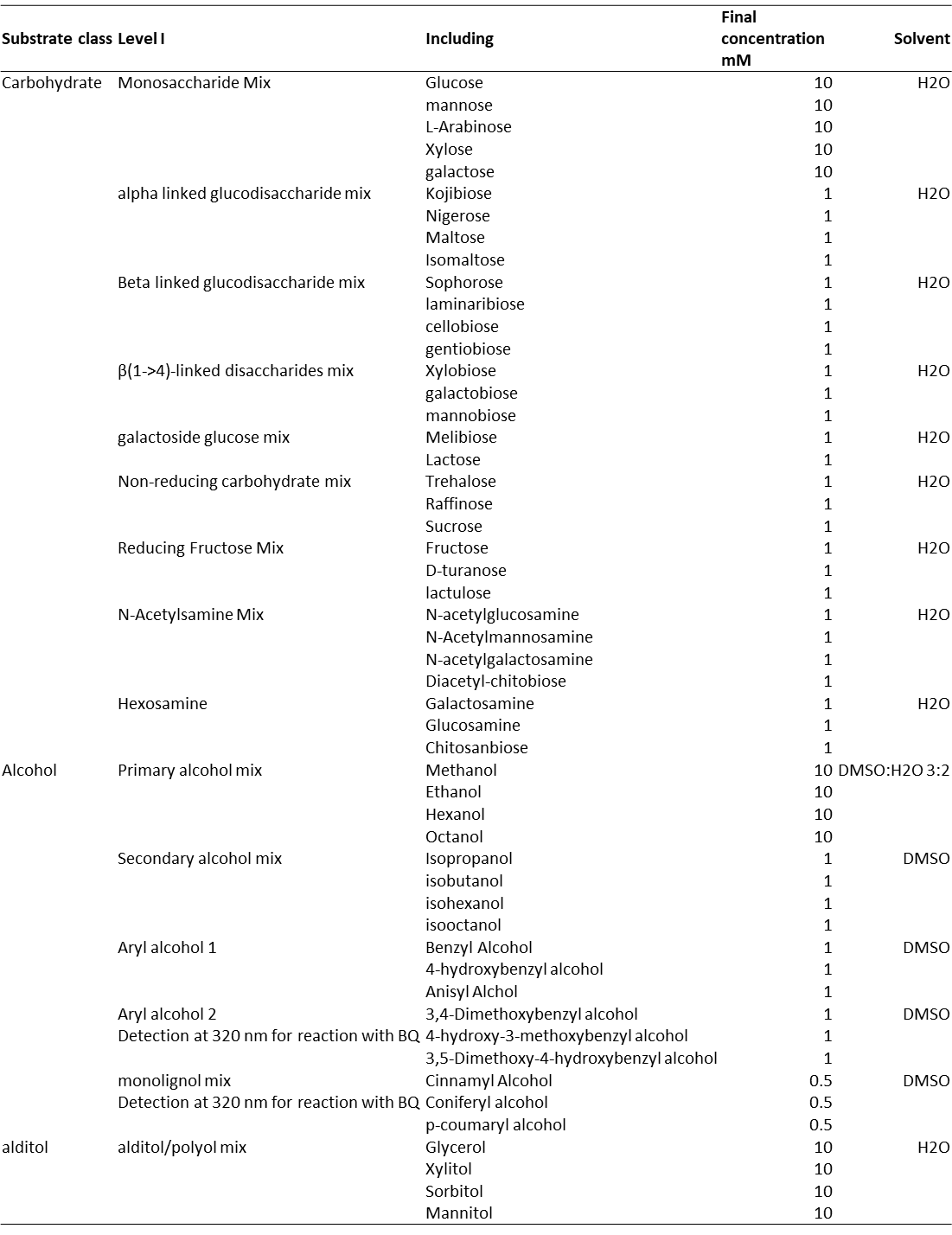
**Table S4. The list of substrates that were tested for the activity assays.

**
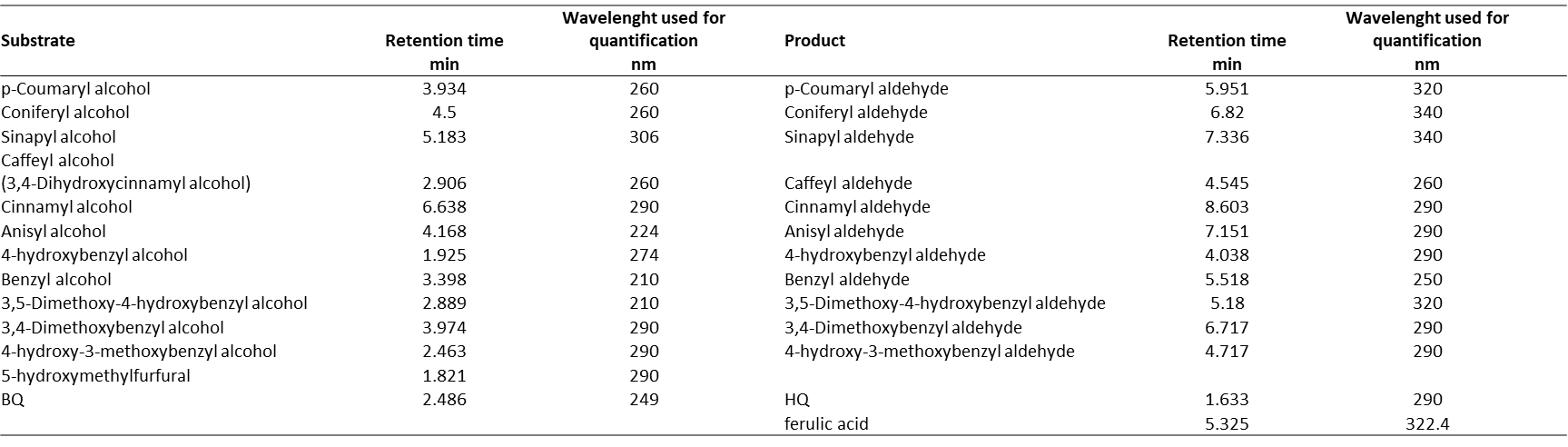
**Table S5. UPLC PDA retention time for each compound and the wavelength used for quantification of each compound

Table S6. Substrate depletion by *Ki*OdhA followed by HPAEC-PAD after 24 h incubation.

|  | Percentage (%) |  | Percentage (%) |
| --- | --- | --- | --- |
| Gentiobiose | 78.6±1.3 | Mannose | BQL |
| Melibiose | 5.1±2.8 | L-arabinose | BQL |
| Glucose | 3.7±1.4 | Isomaltose | BQL |
| Xylose | BQL | Laminaribiose | BQL |

BQL: Below quantification limit.

**
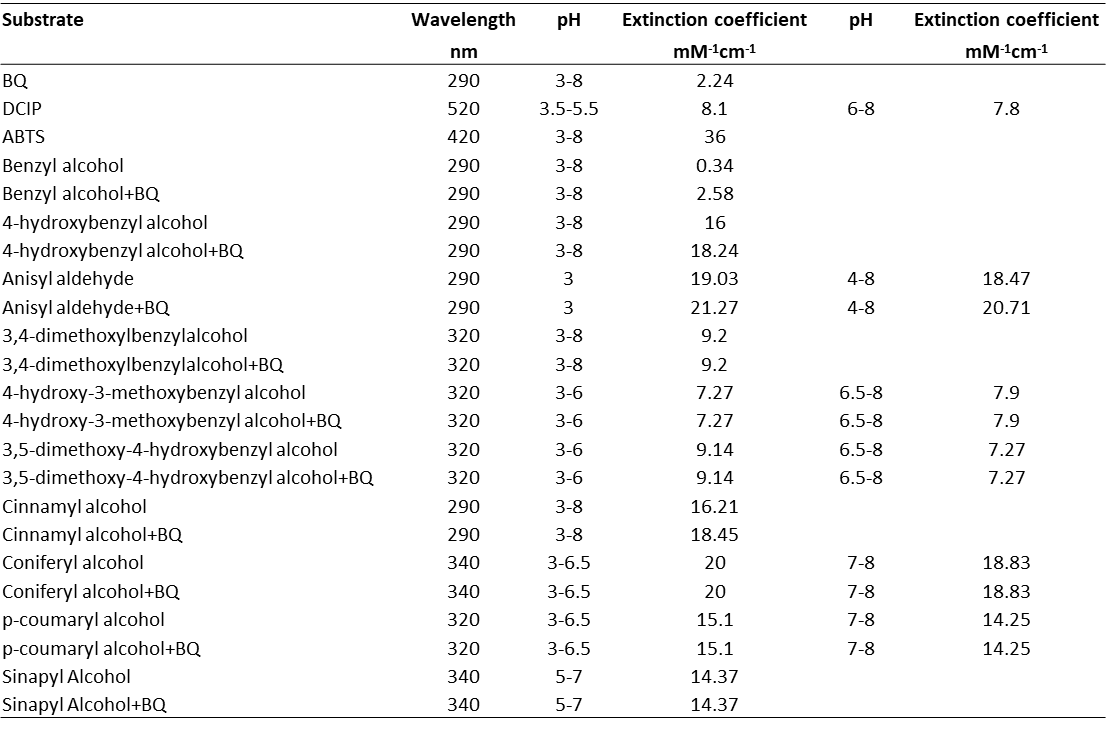
**Table S7. The extinction coefficient and wavelength to be used for the activity assay on aryl alcohols under different pH.


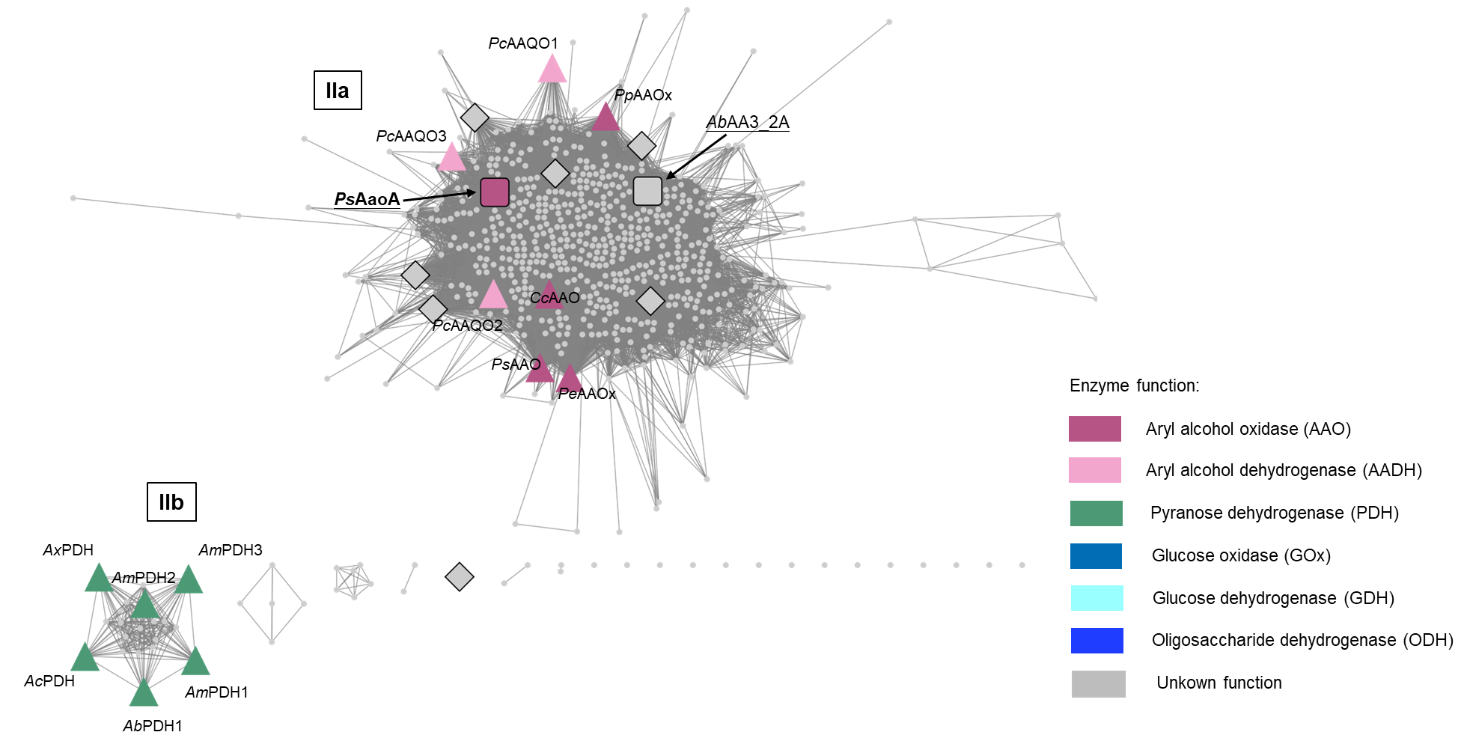


Figure S1. SSN at the cut-off of 470 for the further division of cluster II.


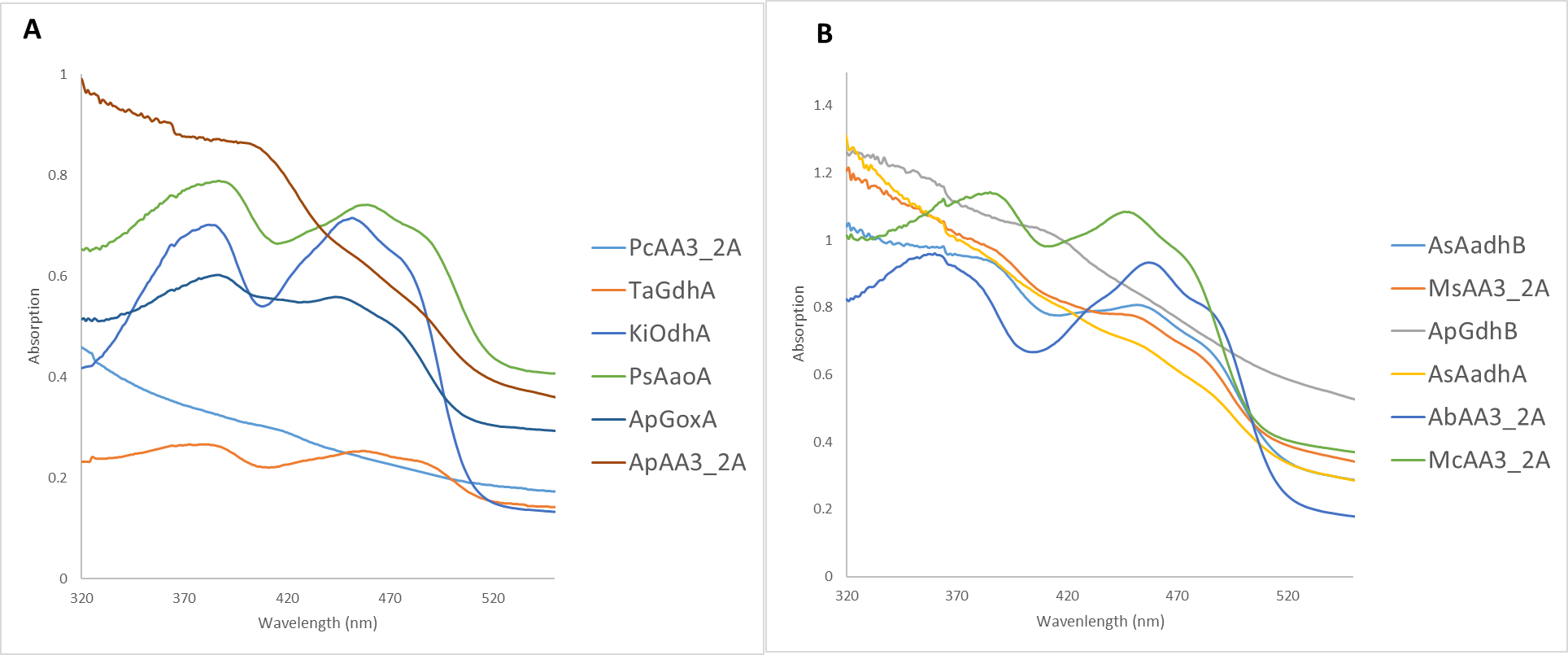


Figure S2. Absorption spectra of the concentrated AA3_2s. The oxidized FAD should have two absorbance maxima at 375-380 nm and at 440-444 nm.


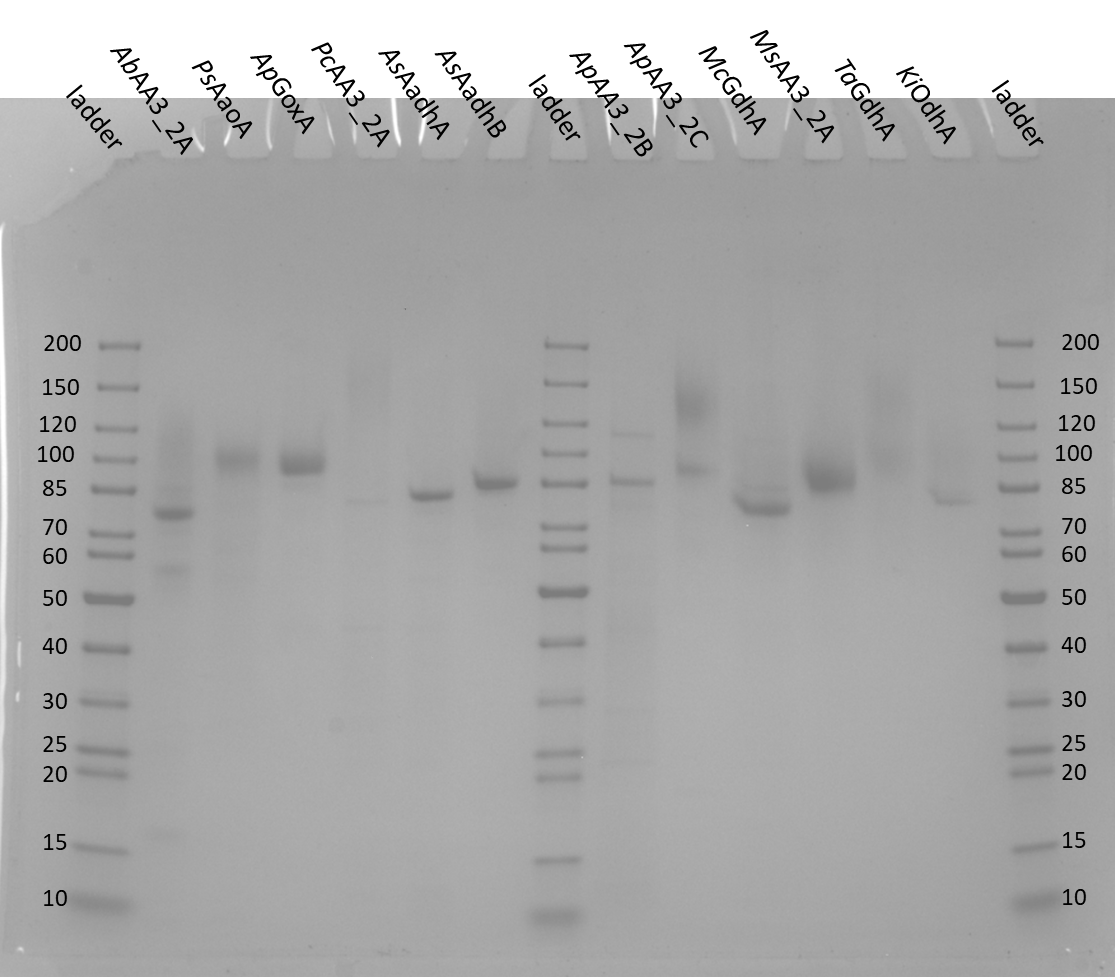


Figure S3. SDS page gel of the successfully produced AA3_2 proteins.


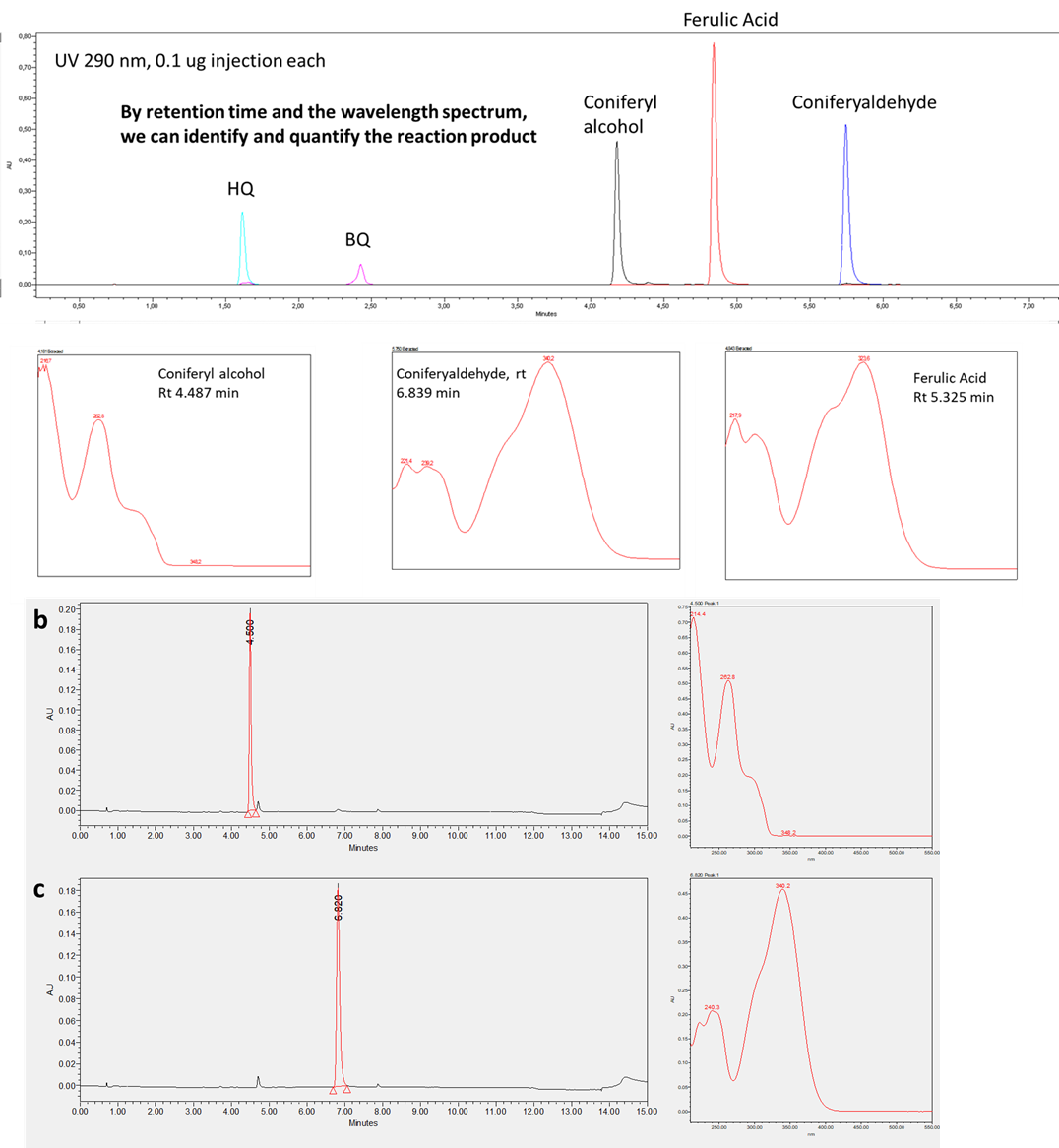


**a**

Figure S4. UPLC-PDA Chromatogram (290 nm) of a) Standards of coniferyl alcohol, coniferaldehyde, ferulic acid, benzoquinone and hydroquinone; b) Coniferyl alcohol after 8 h incubation with boiled *Ps*AaoA at 30 °C; and c) Coniferyl alcohol after *Ps*AaoA oxidation for 8 hour at 30 °C, showing the formation of coniferaldehyde.


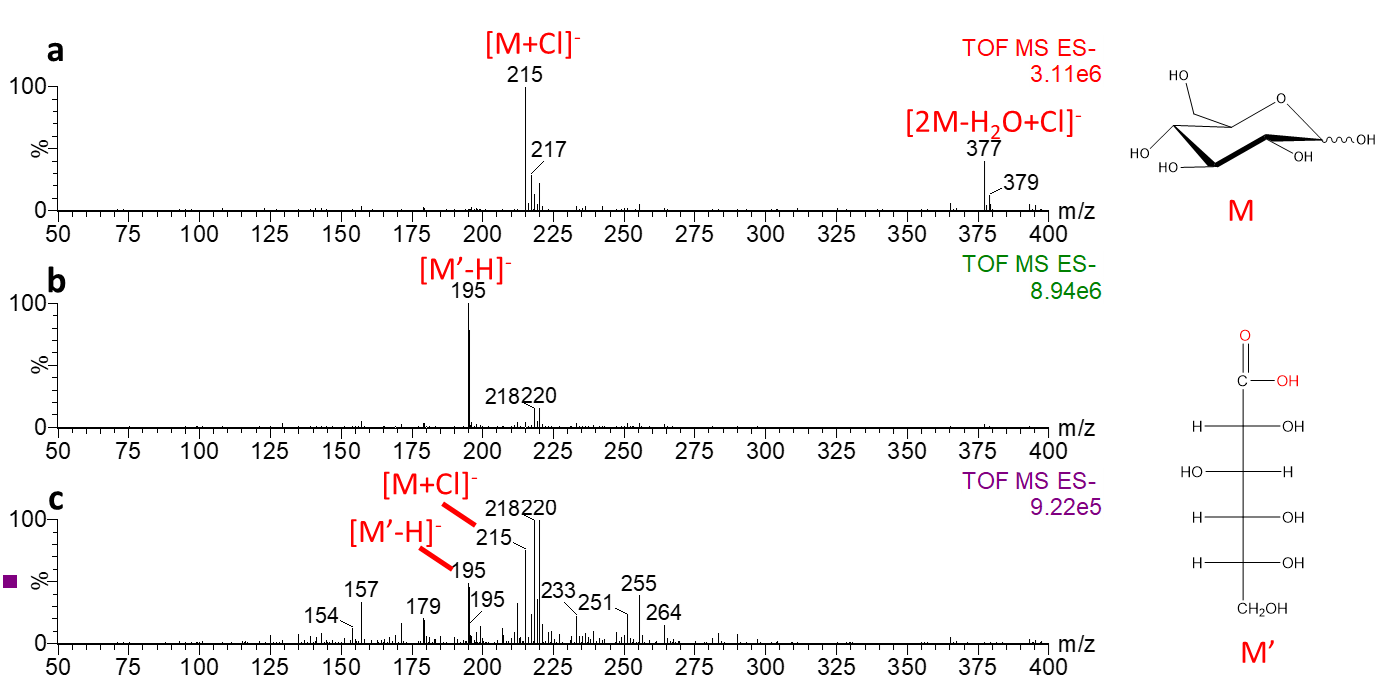


Figure S5. Mass spectra collected in negative ion mode showing a) Glucose b) Glucose after oxidation by *Ap*GoxA and c) Glucose after oxidation by *Ta*GdhA. The additional peaks with m/z ratio between 150 and 265 are background noise derived from the solvents (methanol and water).


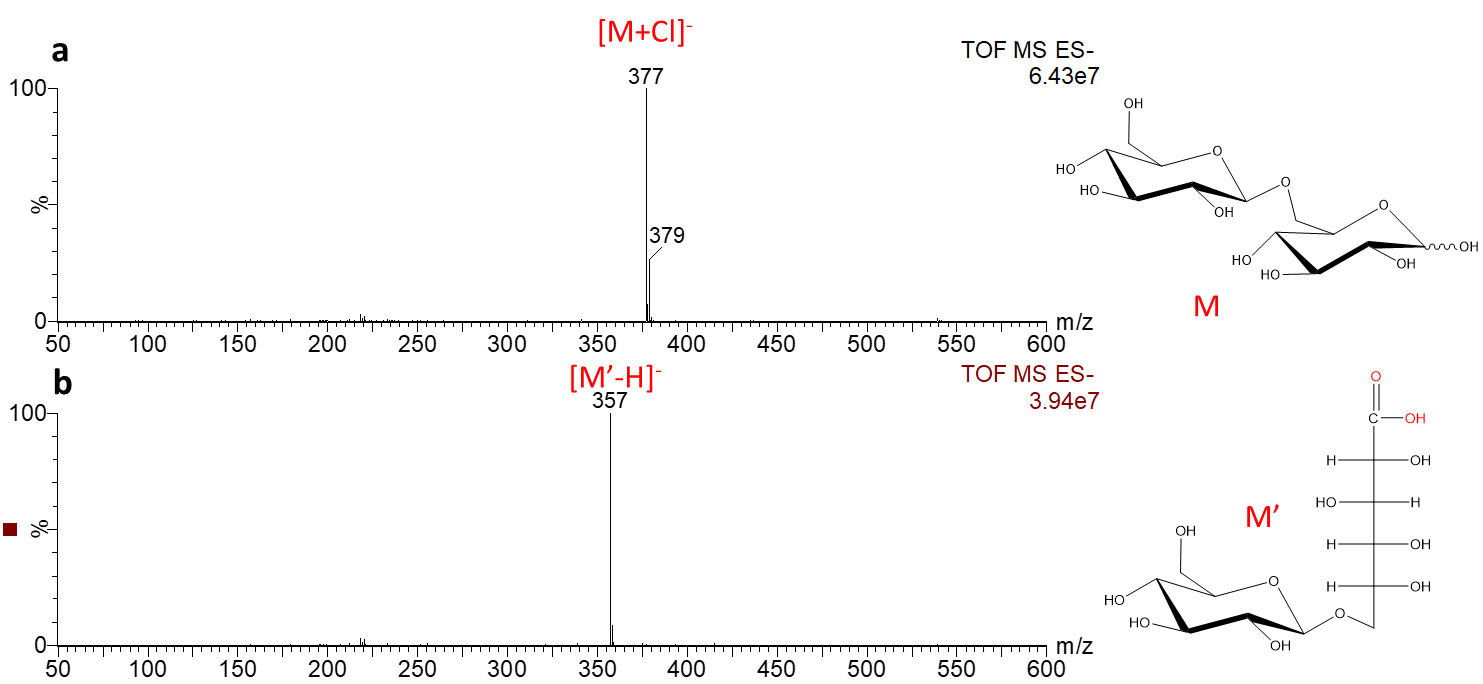


Figure S6. Mass spectra collected in negative ion mode showing a) Gentiobiose and b) Gentiobiose after oxidation by KiOdhA


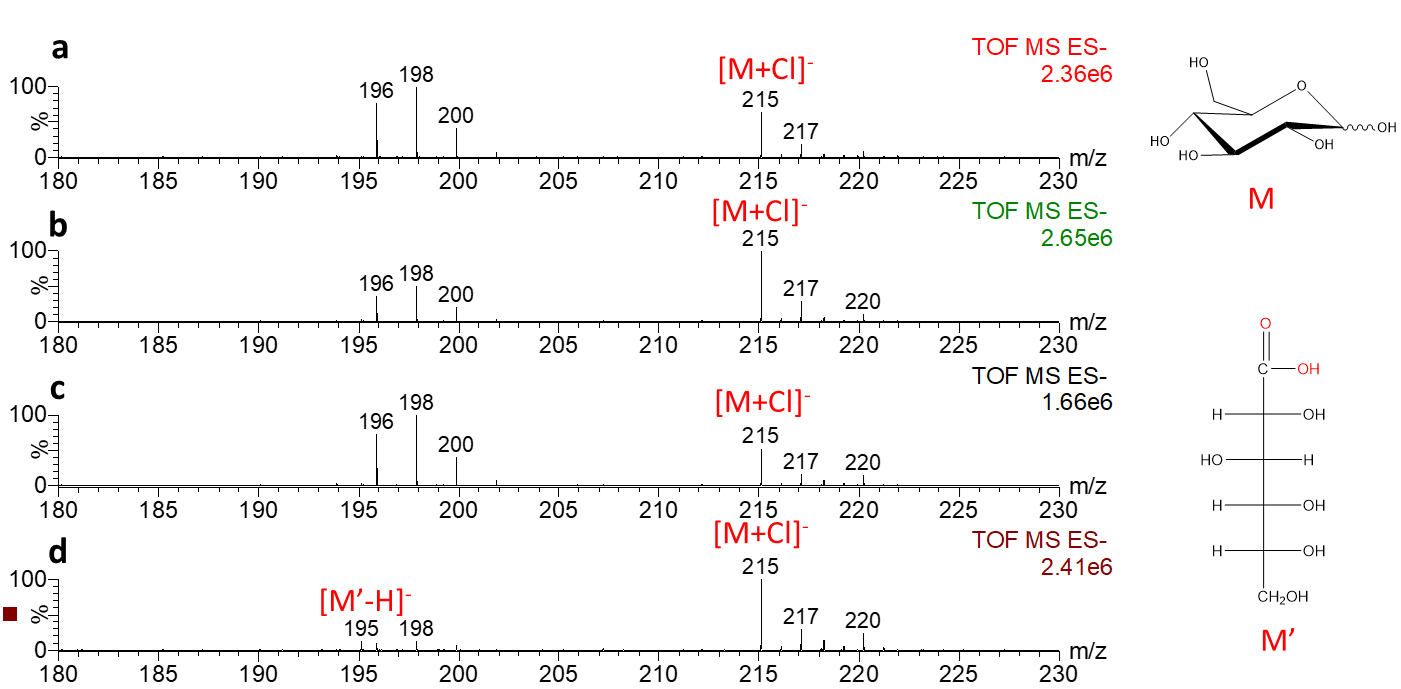


Figure S7. Mass spectra collected in negative ion mode showing a) Glucose; b) Glucose after incubation with *Ap*AA3_2B; c) Glucose after incubation with *Pc*AA3_2A; and d) Glucose after incubation with *Mc*GdhA.


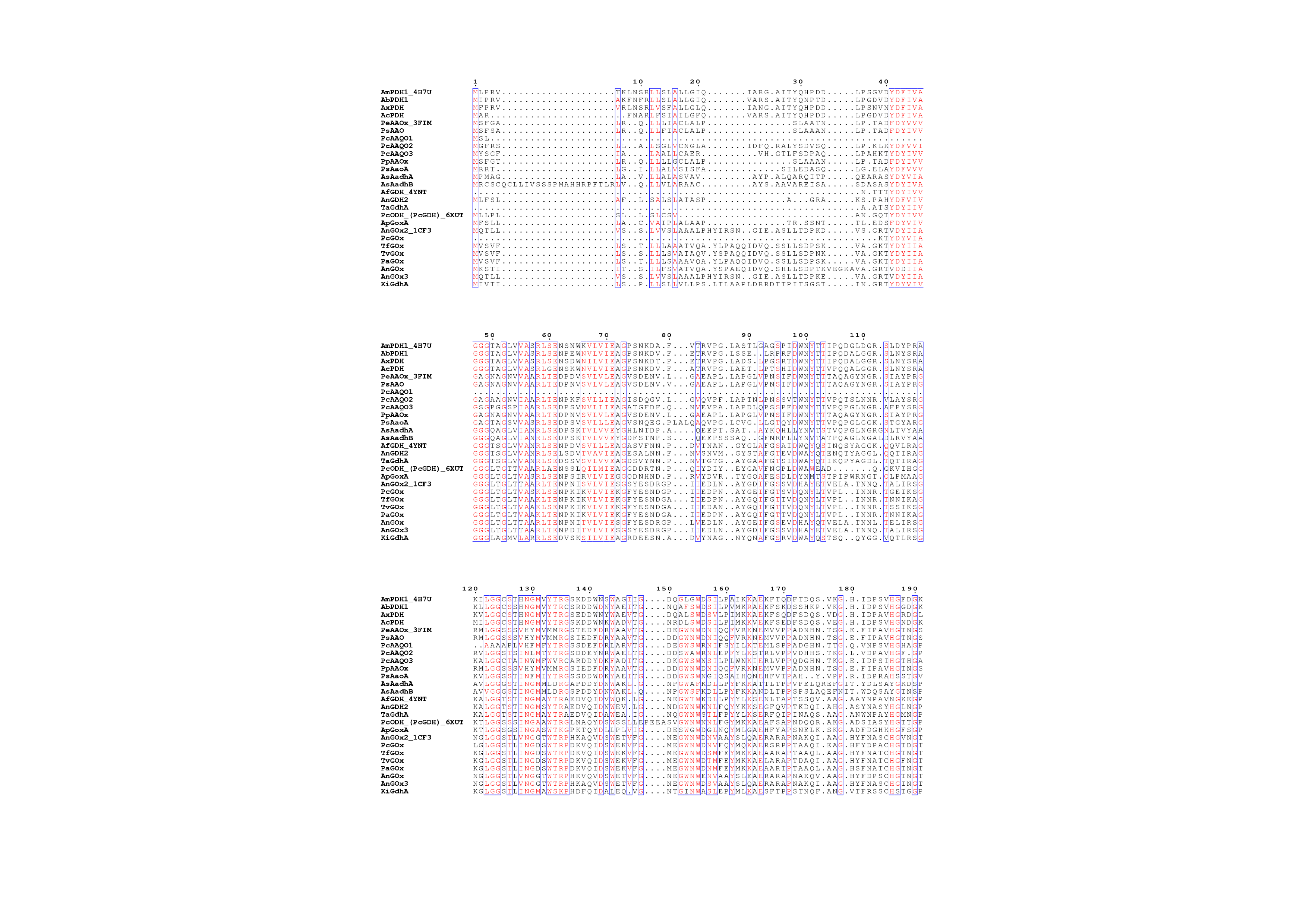

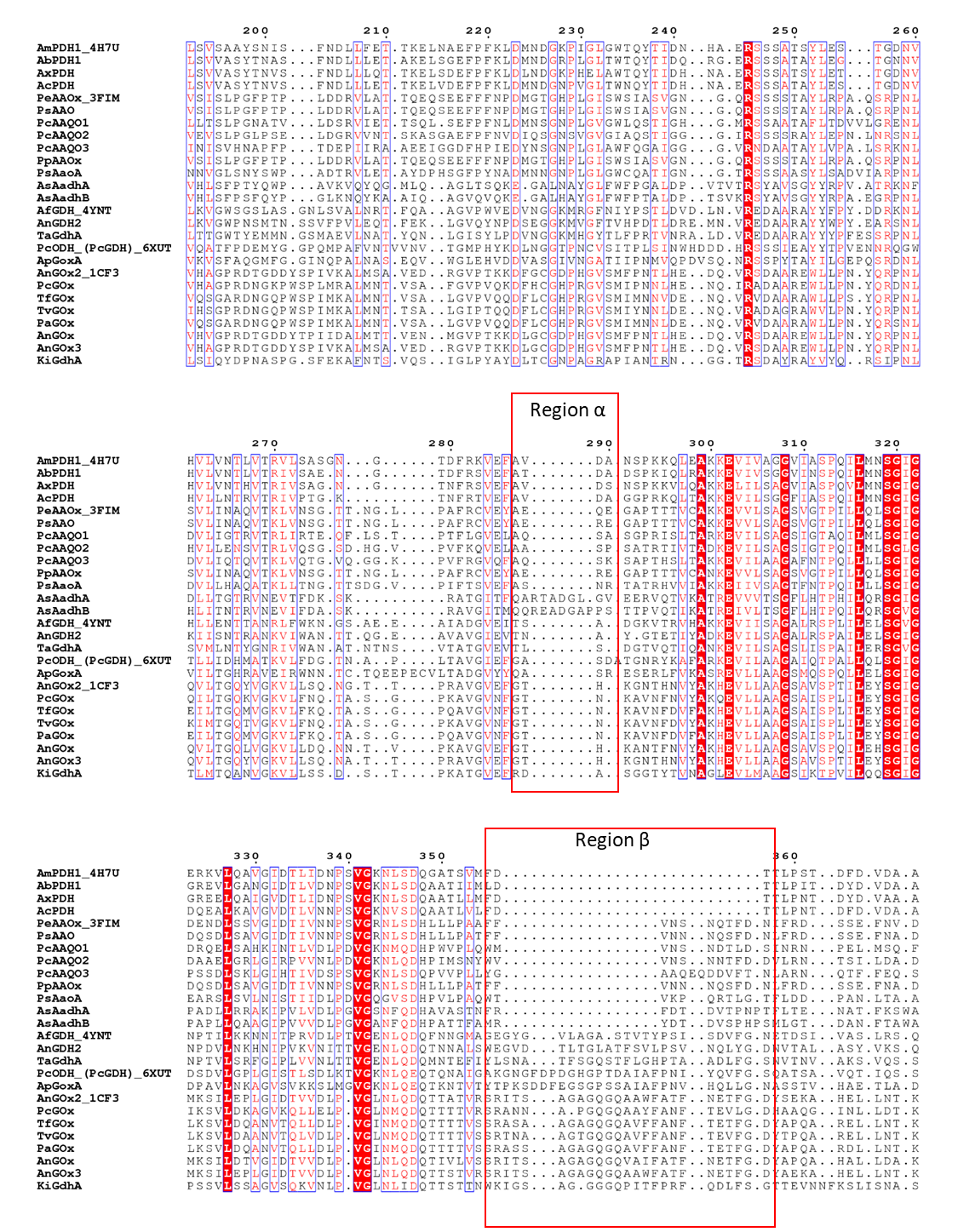

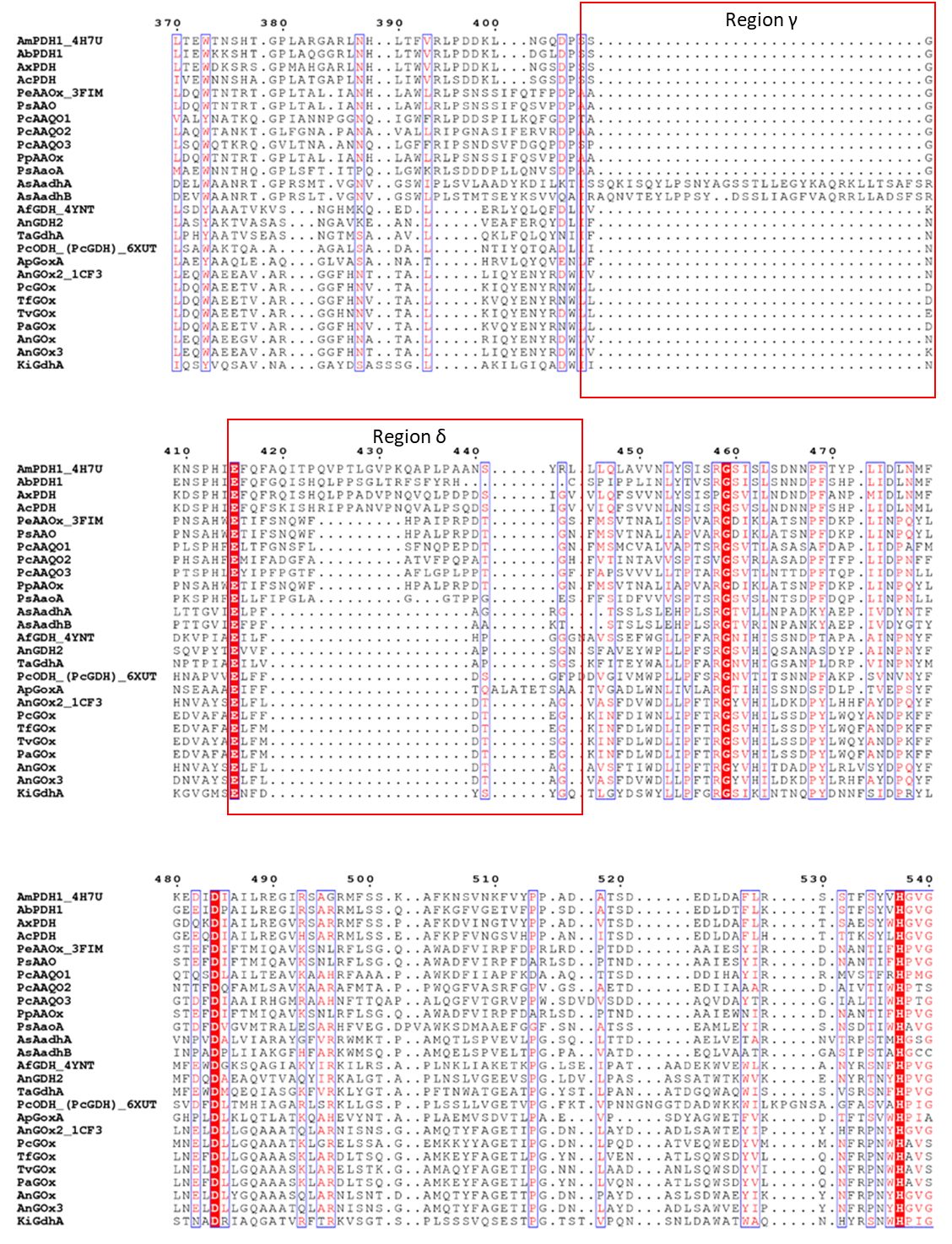

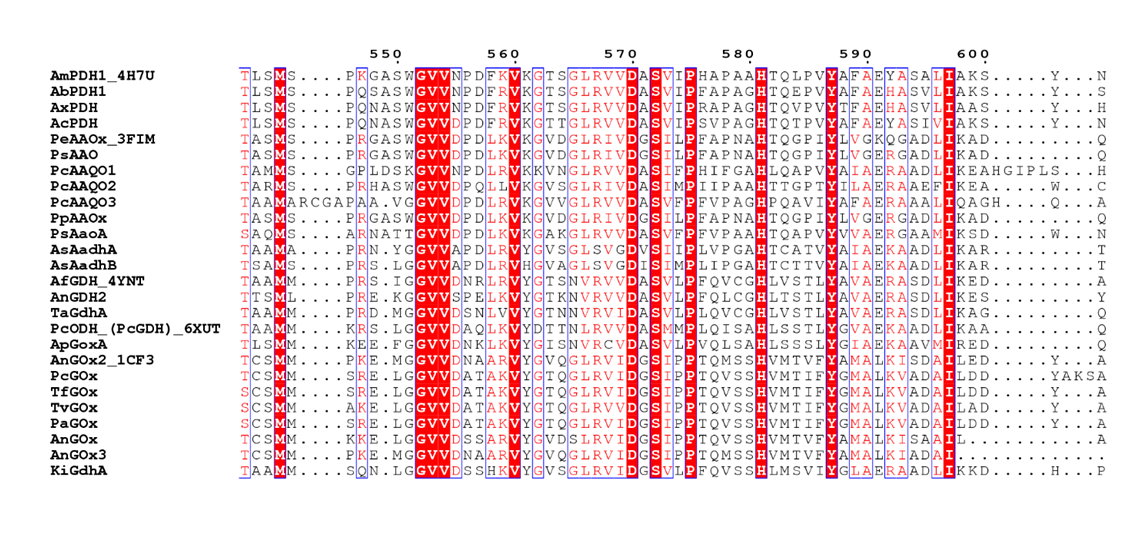


Figure S8. Multiple sequence alignment (MSA) of AA3_2s characterized in this study and some AA3_2s characterized previously. Red boxes show the primary sequence differences between the different enzymes.


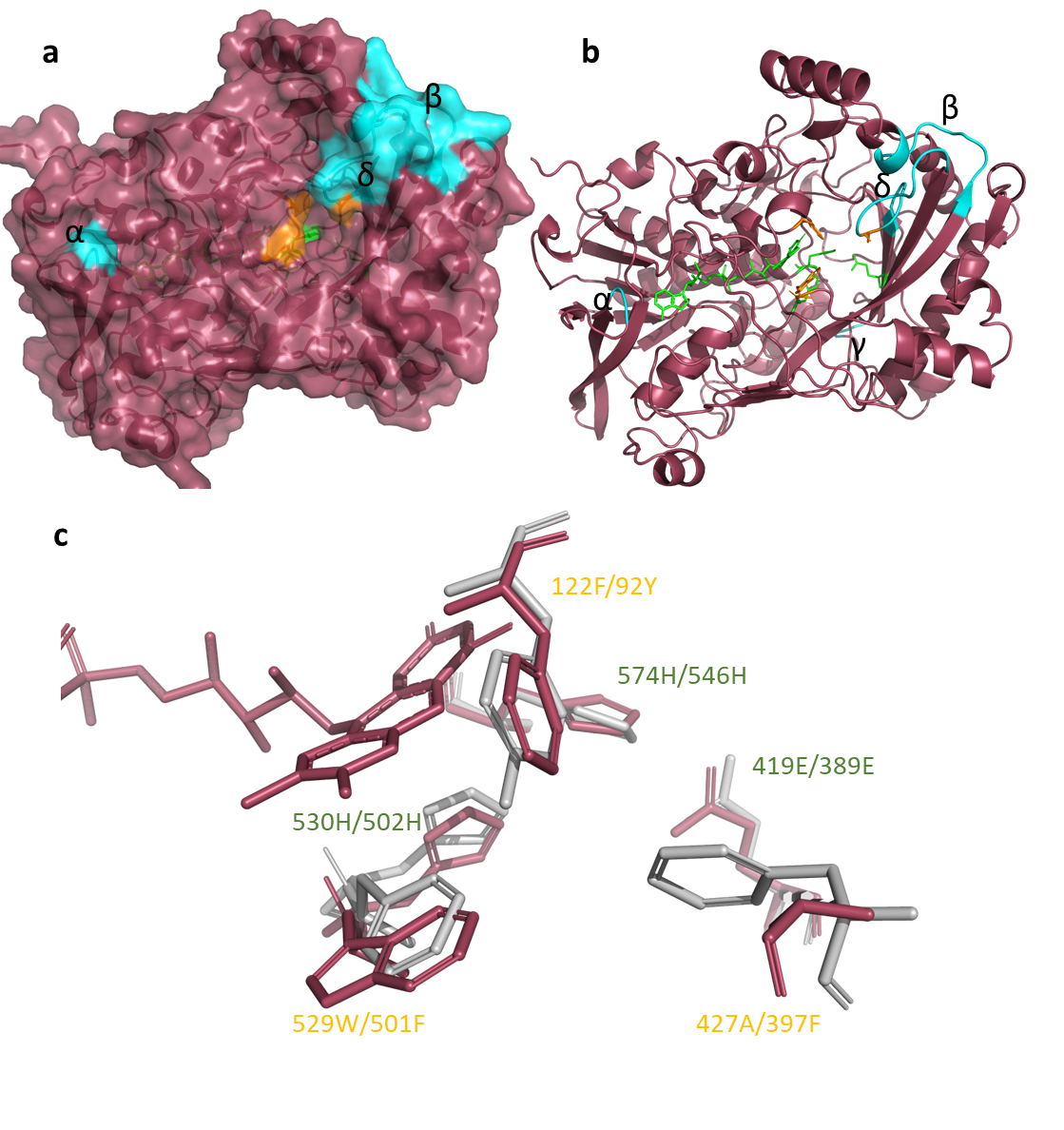


Figure S9. a) Surface and b) ribbon and sticks (active site and FAD) of the AlphaFold homology model of *Ps*AaoA. The FAD and catalytic residues colored in green, hydrophobic residues to form the tunnel to block free access to active site are shown in orange, and the unique motifs identified from MSA are shown in Cyan. c) Alignment for the active site of *Ps*AaoA (Red) and *Pe*AAOx (white, PDB: 3FIM).


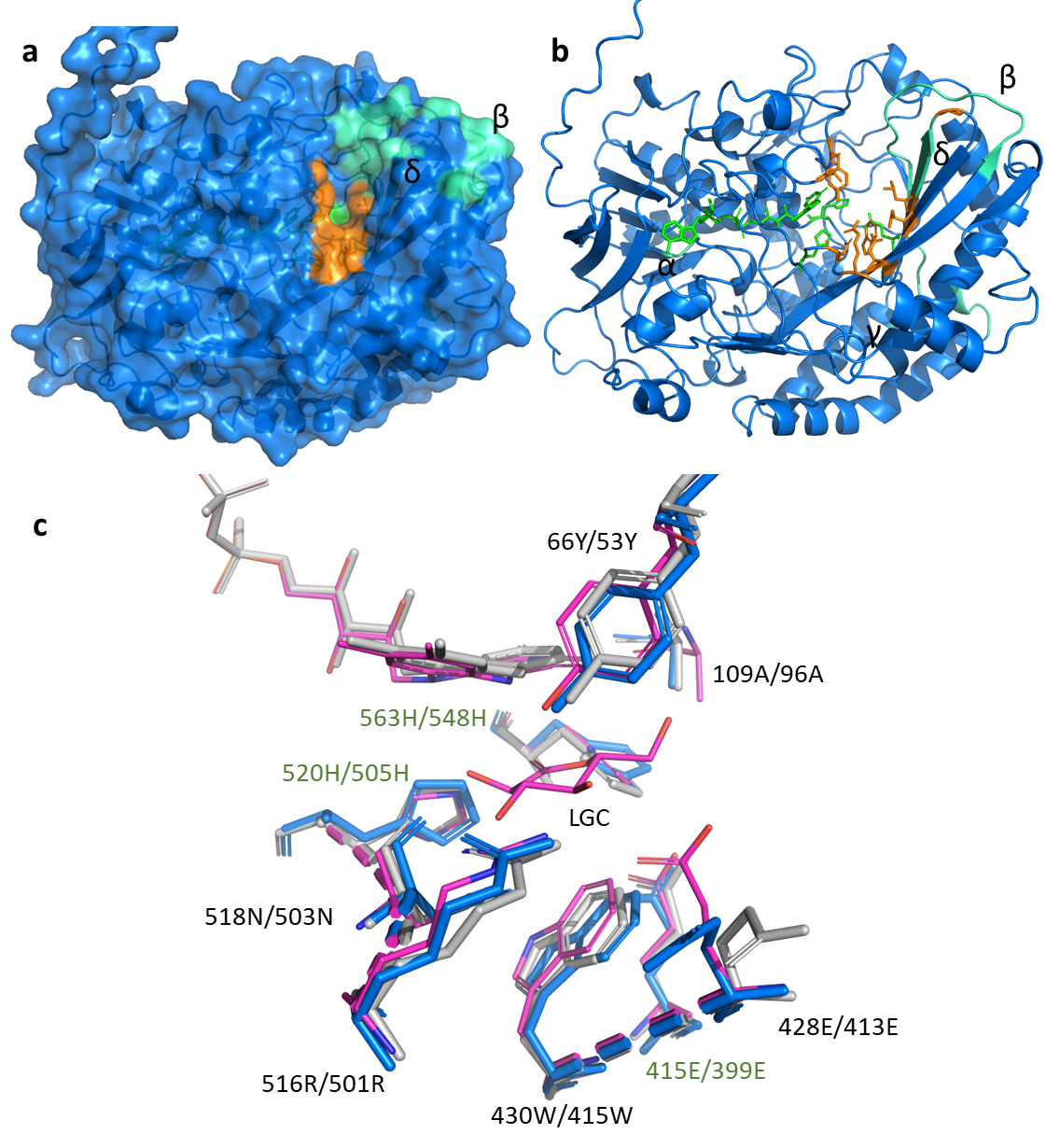


Figure S10. a) Surface and b) ribbon and sticks (active site and FAD) of the AlphaFold homology model of *Ta*GdhA. The FAD and catalytic residues colored in green, residues for substrate binding are shown in orange, and the unique motifs identified from MSA are shown in cyan. c) Alignment for the active site of *Ta*GdhA (blue), *Af*GDH (white, PDB: 4YNT), and *Af*GDH in complex with D-glucono-1,5-lactone (pink, PDB: 4YNU).


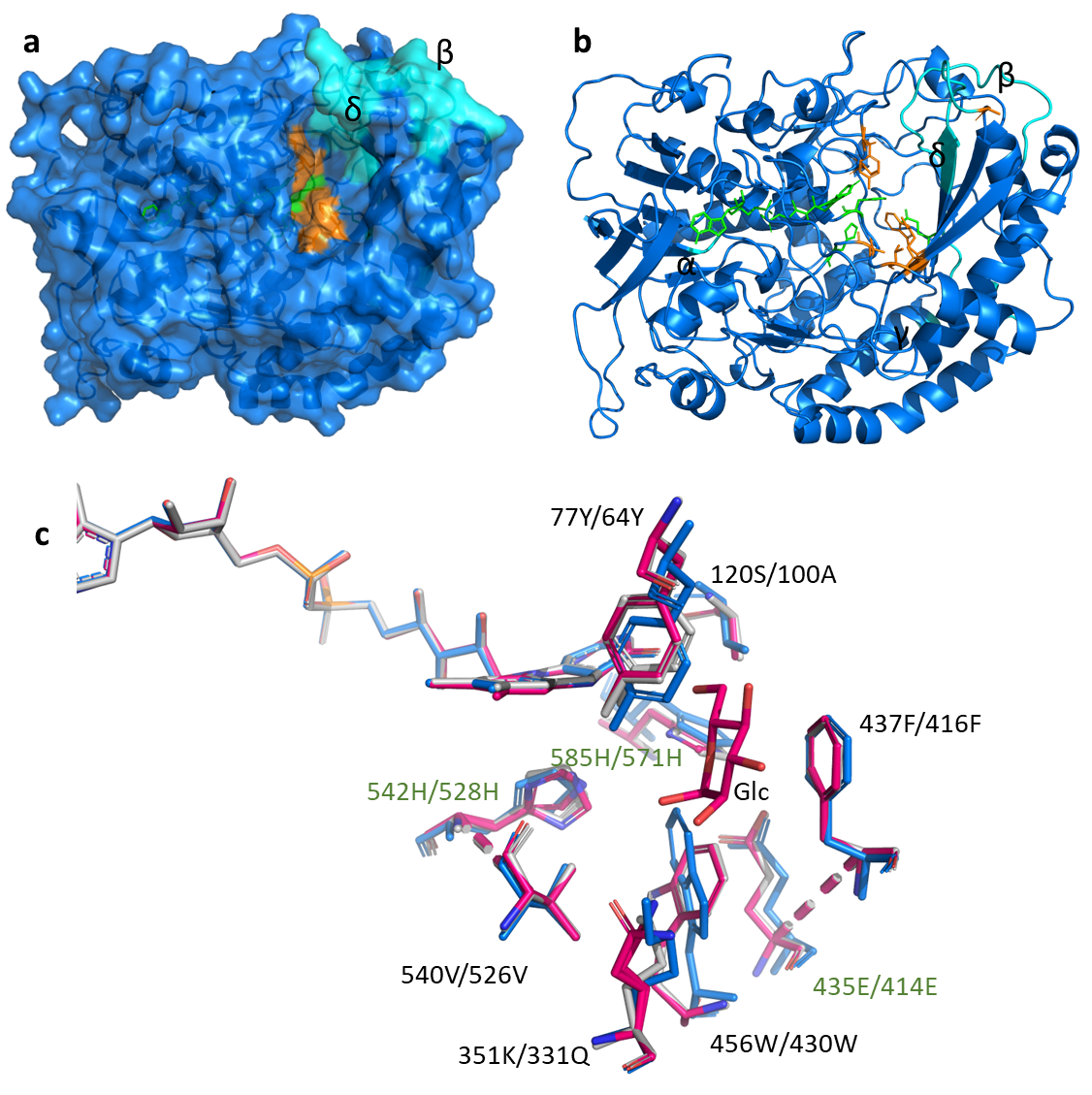


Figure S11. A) Surface and B) ribbon and sticks (active site and FAD) of the AlphaFold homology model of *Ap*GoxA. The FAD and catalytic residues colored in green, residues for substrate binding are shown in orange, and the unique motifs identified from MSA are shown in cyan. C) Alignment for the active site of *Ap*GoxA (blue), *Tc*ODH (white, PDB: 6XUT), and *Tc*ODH in complex with glucose (pink, PDB: 6XUU).


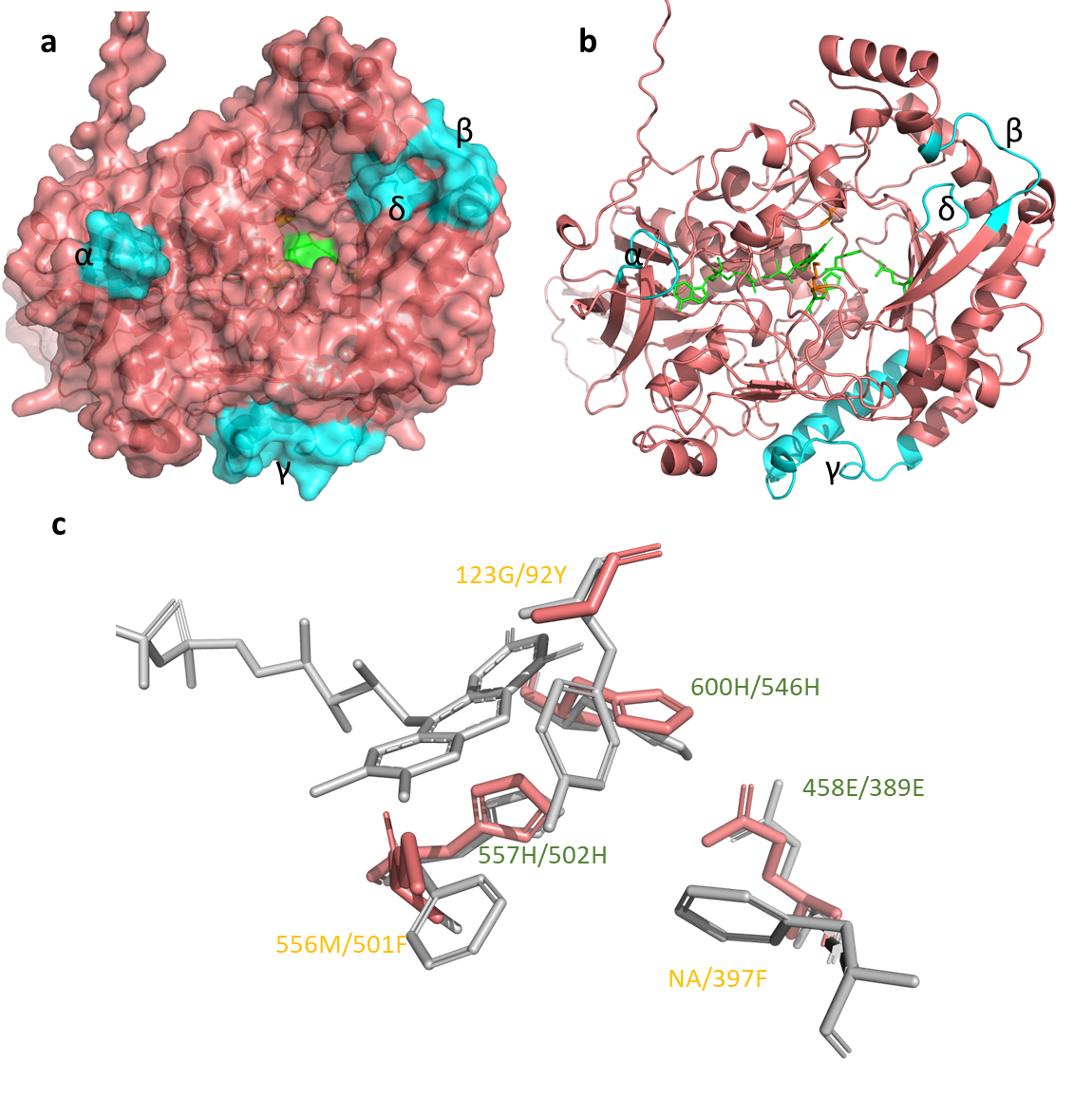


Figure S12. a) Surface and b) ribbon and sticks (active site and FAD) of the AlphaFold homology model of *As*AadhA. The FAD and catalytic residues colored in green and the unique motifs identified from MSA are shown in cyan. c) Alignment for the active site of *As*AadhA (Red) and *Pe*AAO (white, PDB: 3FIM).


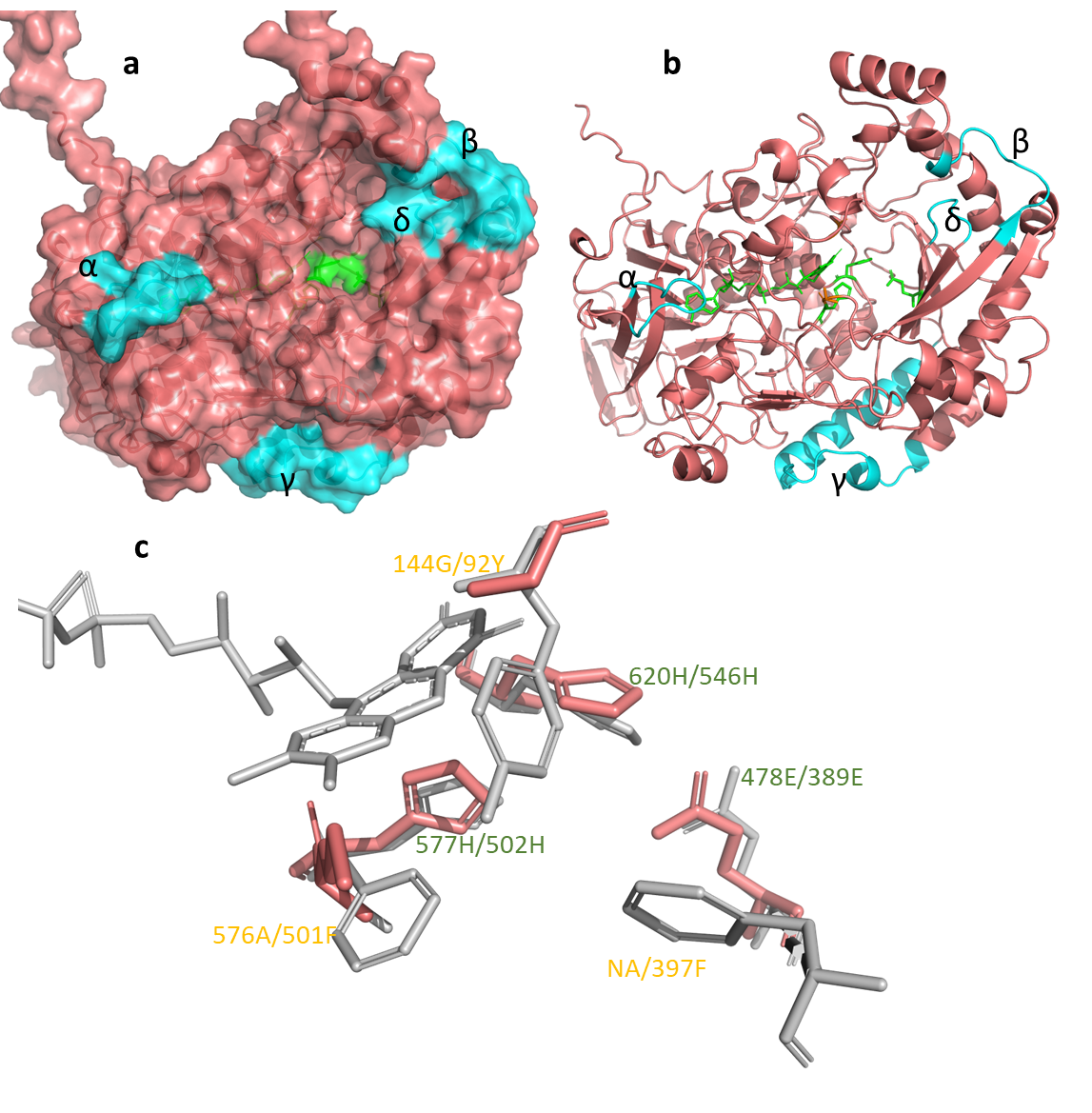


Figure S13. a) Surface and b) ribbon and sticks (active site and FAD) of the AlphaFold homology model of *As*AadhB. The FAD and catalytic residues colored in green and the unique motifs identified from MSA are shown in cyan. c) Alignment for the active site of *As*AadhB (Red) and *Pe*AAO (white, PDB: 3FIM).


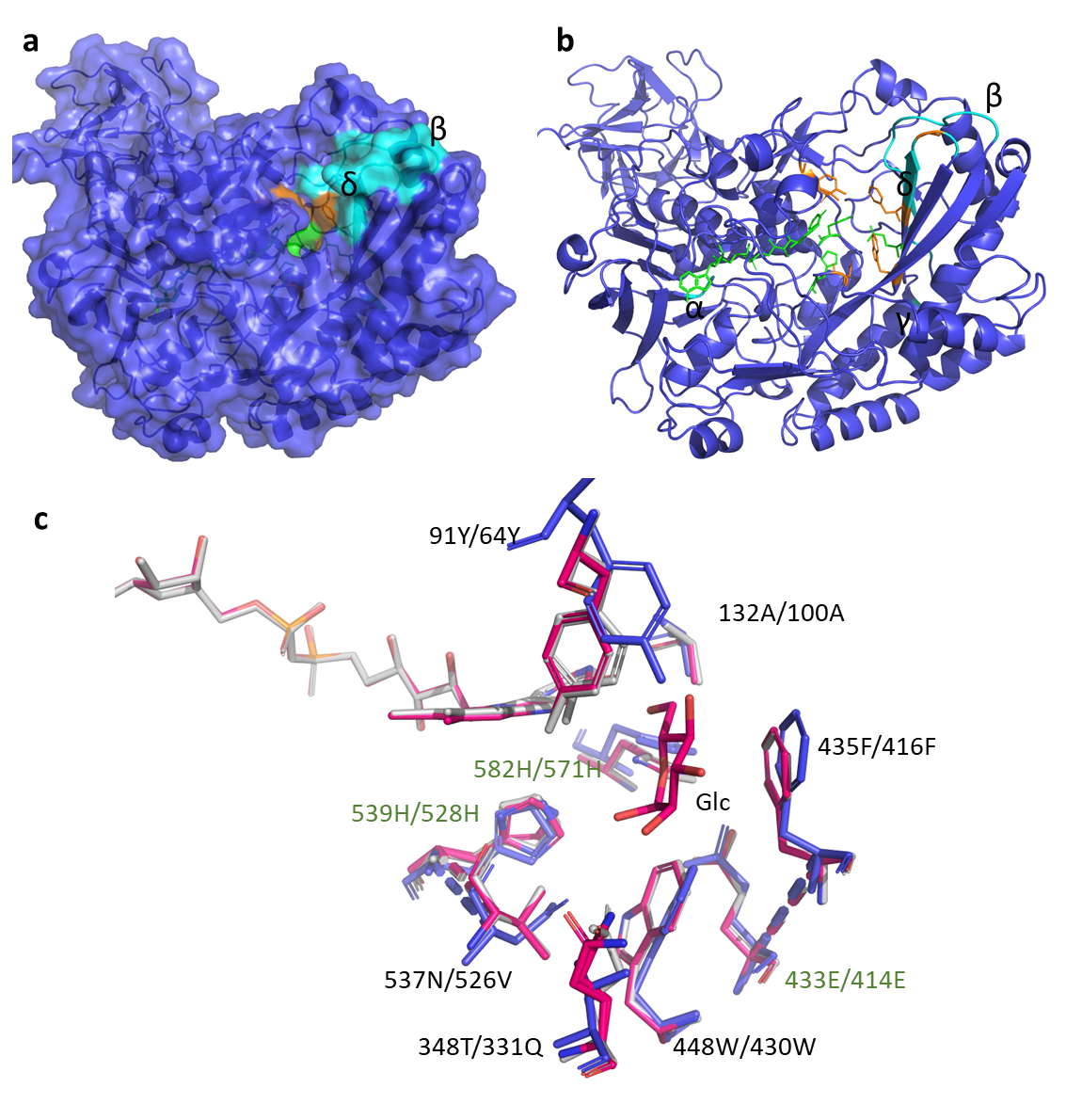


Figure S14. a) Surface and b) ribbon and sticks (active site and FAD) of the AlphaFold homology model of *Ki*OdhA. The FAD and catalytic residues colored in green, residues for substrate binding are shown in orange, and the unique motifs identified from MSA are shown in cyan. c) Alignment for the active site of *Ki*OdhA (blue), *Tc*ODH (white, PDB: 6XUT), and *Tc*ODH in complex with glucose (pink, PDB: 6XUU).


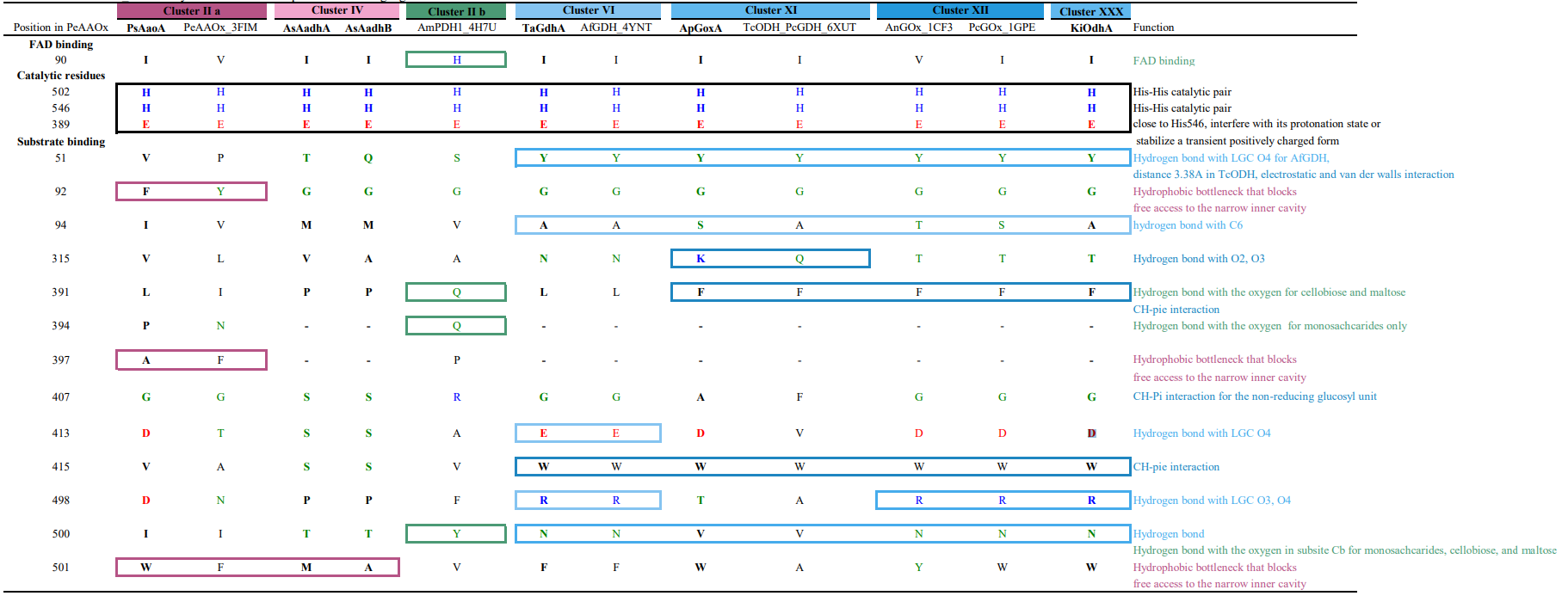


Figure S15. Amino acids and positions within the characterized AA3_2 sequences that are implicated in catalysis and substrate preference.


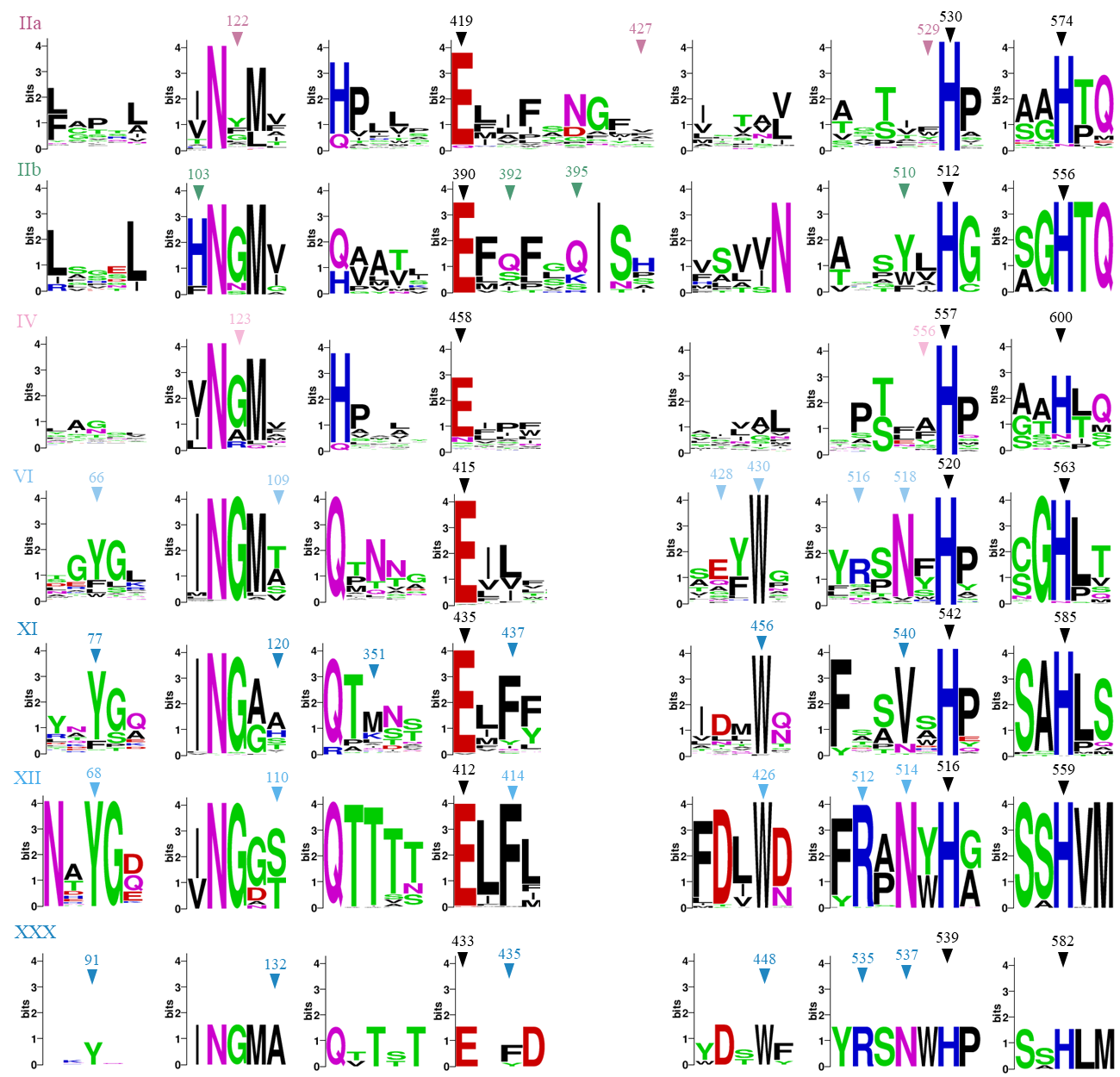


Figure S16. Sequence logos of the active site residues from clades IIa, IIb, IV, VI, XI, XII, and XXX. The amino acid numbering of the sequences is based on *Ps*AaoA for cluster IIa, *Am*PDH1 for cluster IIb, *As*AadhA for cluster IV, *Ta*GdhA for cluster VI, *Ap*GoxA for cluster XI, *An*GOx for cluster XII, and *Ki*OdhA for cluster XXX.

**Reference**

1. Couturier M, Mathieu Y, Li A, Navarro D, Drula E, Haon M, et al. Characterization of a new aryl-alcohol oxidase secreted by the phytopathogenic fungus *Ustilago maydis*. Appl Microbiol Biotechnol. 2016;100:697–706.

2. Ruiz-Dueñas FJ, Ferreira P, Martínez MJ, Martínez AT. In vitro activation, purification, and characterization of *Escherichia coli* expressed aryl-alcohol oxidase, a unique H2O2-producing enzyme. Protein Expr Purif. 2006;45:191–9.

3. Varela E, Böckle B, Romero A, Martínez AT, Martínez MJ. Biochemical characterization, cDNA cloning and protein crystallization of aryl-alcohol oxidase from *Pleurotus pulmonarius*. Biochim Biophys Acta - Protein Struct Mol Enzymol. 2000;1476:129–38.

4. Mathieu Y, Piumi F, Valli R, Aramburu JC, Ferreira P, Faulds CB, et al. Activities of secreted aryl alcohol quinone oxidoreductases from *Pycnoporus cinnabarinus* provide insights into fungal degradation of plant biomass. Appl Environ Microbiol. 2016;82:2411–23.

5. Galperin I, Javeed A, Luig H, Lochnit G, Rühl M. An aryl-alcohol oxidase of *Pleurotus sapidus*: heterologous expression, characterization, and application in a 2-enzyme system. Appl Microbiol Biotechnol. 2016;100:8021–30.

6. Tamaru Y, Umezawa K, Yoshida M. Characterization of an aryl-alcohol oxidase from the plant saprophytic basidiomycete *Coprinopsis cinerea* with broad substrate specificity against aromatic alcohols. Biotechnol Lett. 2018;40:1077–86.

7. Kittl R, Sygmund C, Halada P, Volc J, Divne C, Haltrich D, et al. Molecular cloning of three pyranose dehydrogenase-encoding genes from *Agaricus meleagris* and analysis of their expression by real-time RT-PCR. Curr Genet. 2008;53:117–27.

8. Staudigl P, Krondorfer I, Haltrich D, Peterbauer CK. Pyranose dehydrogenase from *Agaricus campestris* and *Agaricus xanthoderma*: Characterization and applications in carbohydrate conversions. Biomolecules. 2013;3:535–52.

9. Volc J, Sedmera P, Halada P, Přikrylová V, Haltrich D. Double oxidation of D-xylose to D-glycero-pentos-2,3-diulose (2,3-diketo-D-xylose) by pyranose dehydrogenase from the mushroom *Agaricus bisporus*. Carbohydr Res. 2000;329:219–25.

10. Graf MMH, Weber S, Kracher D, Kittl R, Sygmund C, Ludwig R, et al. Characterization of three pyranose dehydrogenase isoforms from the litter-decomposing basidiomycete *Leucoagaricus meleagris* (syn. *Agaricus meleagris*). Appl Microbiol Biotechnol. 2017;101:2879–91.

11. Kadowaki MAS, Higasi PMR, de Godoy MO, de Araújo EA, Godoy AS, Prade RA, et al. Enzymatic versatility and thermostability of a new aryl-alcohol oxidase from *Thermothelomyces thermophilus* M77. Biochim Biophys Acta - Gen Subj. 2020;1864:129681.

12. Wijayanti SD, Sützl L, Duval A, Haltrich D. Characterization of fungal FAD-dependent AA3_2 glucose oxidoreductases from hitherto unexplored phylogenetic clades. J Fungi. 2021;7.

13. Sygmund C, Klausberger M, Felice AK, Ludwig R. Reduction of quinones and phenoxy radicals by extracellular glucose dehydrogenase from *Glomerella cingulata* suggests a role in plant pathogenicity. Microbiology. 2011;157:3203–12.

14. Yoshida H, Sakai G, Mori K, Kojima K, Kamitori S, Sode K. Structural analysis of fungus-derived FAD glucose dehydrogenase. Sci Rep. 2015;5:1–13.

15. Mori K, Nakajima M, Kojima K, Murakami K, Ferri S, Sode K. Screening of Aspergillus-derived FAD-glucose dehydrogenases from fungal genome database. Biotechnol Lett. 2011;33:2255–63.

16. Cerutti G, Gugole E, Montemiglio LC, Turbé-Doan A, Chena D, Navarro D, et al. Crystal structure and functional characterization of an oligosaccharide dehydrogenase from *Pycnoporus cinnabarinus* provides insights into fungal breakdown of lignocellulose. Biotechnol Biofuels. 2021;14:1–18.

17. Frederick KR, Tung J, Emerick RS, Masiarz FR, Chamberlain SH, Vasavada A, et al. Glucose oxidase from Aspergillus niger. Cloning, gene sequence, secretion from *Saccharomyces cerevisiae* and kinetic analysis of a yeast-derived enzyme. J Biol Chem. Elsevier; 1990;265:3793–802.

18. Hatzinikolaou DG, Hansen OC, Macris BJ, Tingey A, Kekos D, Goodenough P, et al. A new glucose oxidase from *Aspergillus niger*: Characterization and regulation studies of enzyme and gene. Appl Microbiol Biotechnol. 1996;46:371–81.

19. Kiess M, Hecht HJ, Kalisz HM. Glucose oxidase from *Penicillium amagasakiense* Primary structure and comparison with other glucose-methanol-choline (GMC) oxidoreductases. Eur J Biochem. 1998;252:90–9.

20. Gao Z, Li Z, Zhang Y, Huang H, Li M, Zhou L, et al. High-level expression of the *Penicillium notatum* glucose oxidase gene in *Pichia pastoris* using codon optimization. Biotechnol Lett. 2012;34:507–14.

21. Pulci V, D’Ovidio R, Petruccioli M, Federici F. The glucose oxidase of *Penicillium variabile* P16: Gene cloning, sequencing and expression. Lett Appl Microbiol. 2004;38:233–8.

22. Murray FR, Llewellyn DJ, Peacock WJ, Dennis ES. Isolation of the glucose oxidase gene from *Talaromyces flavus* and characterisation of its role in the biocontrol of *Verticillium dahliae*. Curr Genet. 1997;32:367–75.
